# Occupational health and safety standards compliance among construction workers in West Africa: A systematic review of available evidence from Ghana and Nigeria

**DOI:** 10.1371/journal.pgph.0006724

**Published:** 2026-11-13

**Authors:** Kimblyn Anim, Saphiel Osei Poku, Glypsy Agyiri-Kyereme

**Affiliations:** 1 Department of Urban Roads, Ministry of Roads and Highways, Kumasi, Ashanti Region, Ghana; 2 Department of Optometry and Visual Science, Faculty of Biosciences, College of Science, Kwame Nkrumah University of Science and Technology, Kumasi, Ghana; 3 Faculty of Health and Medical Sciences, Asante Akyem Campus, Presbyterian University, Agogo, Ghana; 4 Nyinahin Community Hospital, Nyinahin, Ashanti Region, Ghana; Universiti Kuala Lumpur Royal College of Medicine Perak, MALAYSIA

## Abstract

The construction industry remains one of the most dangerous sectors globally, with West African workers facing particular risks due to labour-intensive methods and limited regulatory enforcement. No previous systematic review has synthesized evidence on compliance with occupational health and safety (OHS) standards among construction workers in West Africa. We searched PubMed, IEEE Xplore, EBSCOhost, Science Direct and Google Scholar for quantitative studies published from June 2001 to August 18, 2026. Studies required extractable data on occupational health and safety compliance outcomes. Quality assessment used the JBI critical appraisal tool for cross-sectional studies. Studies rated as high risk of bias were excluded. Twelve cross-sectional studies met inclusion criteria: seven from Ghana and five from Nigeria. No eligible low- or moderate-risk studies from the other 14 West African countries were identified through this review. PPE use, access and provision varied substantially with helmet use 16.9%, hard-hat use 34.1% and respirator use of 1.7% during relevant tasks. 54.5% respondents in a Nigerian study reported that they did not wear a mask for silica-dust exposure. Welfare facilities were severely neglected, with flushable toilets on only 14.3% of sites. Safety knowledge and training were often more favourable than behavioural measures, but these constructs were not treated as equivalent to compliance. A study found that lack of safety training and inconsistent PPE use were associated with occupational injury, but the causality was not established. Factors associated with poorer compliance included younger age, lower educational level, casual employment, physical barriers and the absence of a construction‑specific regulatory framework. Evidence from Ghana and Nigeria identifies heterogeneous gaps across OHS compliance-related domains, particularly selected PPE practices, organisational implementation and welfare-facility provision. Knowledge, training, PPE availability and PPE use should not be interpreted as interchangeable compliance measures. Standardised multicountry observational studies and evaluations of proposed interventions are needed.

Trial registration

This systematic review followed PRISMA guidelines and was registered with PROSPERO (CRD420250649740).

## Introduction

Occupational health and safety (OHS) encompasses the prevention of work-related injury and disease and the adaptation of work, equipment and environment to protect workers. Ergonomic design, hazard control, safe work systems and organisational safety practices are important components of this field [1–10]. Globally, occupational accidents and diseases remain substantial public-health and economic burdens, and construction is consistently identified as a high-risk sector because workers are exposed to falls, moving equipment, noise, dusts, chemicals, manual handling, awkward postures and changing work environments [11–18].

Construction OHS depends not only on the existence of rules but also on how those rules are implemented by employers, regulators and workers. OHS management systems commonly emphasise legal compliance, appropriate standards, risk control, training, supervision and continuous improvement [19–24]. Nevertheless, serious injuries, disability and deaths continue to occur, with important direct and indirect costs to workers, families, employers and health systems [25–27]. Compliance therefore has public health significance because failures in basic protective practices can increase exposure to preventable hazards, particularly among casual and informal workers who may have limited bargaining power or access to occupational-health services.

African construction industries face additional implementation challenges related to regulatory capacity, informality, resource constraints and uneven access to OHS expertise [28–33]. West Africa was selected as the review scope because construction labour markets across the subregion share rapid urbanisation, substantial informal employment and cross-border economic linkages, while national OHS governance arrangements remain heterogeneous. This combination makes regional evidence mapping useful: shared patterns can inform broader worker-protection priorities, while differences between national systems can identify where context-specific policy responses are required. The review defined West Africa using the 16 countries in the United Nations M49 Western Africa grouping rather than equating the region with current ECOWAS membership.

Existing scholarship does not make the present synthesis redundant. Reviews have examined broad construction safety and health research in developing countries [34], factors associated with occupational injuries in African high-risk industries, and challenges that undermine construction health and safety regulation across selected African countries [35]. Earlier work has also reviewed regulatory compliance in individual settings such as Nigeria. These reviews address research trends, injuries, regulatory barriers or national regulatory systems rather than systematically extracting and comparing quantitative worker and site-level compliance-related measures across West Africa. A synthesis gap therefore remains in four areas: the measurement of behavioural compliance, organisational and regulatory implementation, safety knowledge and training, and welfare-facility provision; the factors statistically associated with these outcomes, the geographical distribution of the evidence and the distinction between strategies that have merely been proposed and those actually tested.

The aim of this systematic review was to synthesize available quantitative evidence on OHS compliance-related outcomes among construction workers in West Africa. Specifically, the review sought to: (1) characterize findings across PPE use, access and provision; regulatory and organisational implementation; safety knowledge, awareness and training; and provision and use of welfare facilities and services; (2) identify individual, work-related, organisational and regulatory factors associated with OHS compliance-related outcomes; and (3) summarize strategies reported or proposed by the included studies for improving OHS compliance. These objectives were intended to support public health decision-making by identifying measurable gaps in worker protection, clarifying where the evidence is strongest or absent, and highlighting priorities for regulation, surveillance and intervention research.

## Methods

### Protocol and registration

This systematic review followed the guidelines established by the Preferred Reporting Items for Systematic Reviews and Meta-analyses (PRISMA) [36,37]. The systematic review was registered on 24 February 2025 with the International Prospective Register of Systematic Reviews (PROSPERO) (CRD420250649740). The review protocol is available at https://www.crd.york.ac.uk/PROSPERO/view/CRD420250649740. The registered review protocol is provided in S1 Text, and the completed PRISMA 2020 checklist in S1 PRISMA Checklist.

### Eligibility criteria

The PECOS framework guided the selection of literature for eligibility for this study. The PECOS acronym stands for population, exposure, comparator, outcome and study design. Only studies that met the criteria specified in PECOS were included. Table 1 outlines the PECOS criteria used. To focus on studies conducted after the International Labour Organization’s non-binding global guidance on occupational safety and health Management Systems [38], the timeframe of the articles was selected from June 2001 to August 18, 2026. We included only quantitative study designs as the primary aim of this review requires numerical estimates that can be compared across studies. We also only included studies in English. Qualitative, mixed methods studies without separable quantitative data, reviews, editorials, protocols, case studies and non-peer-reviewed reports without original data were excluded. Studies rated as having a high risk of bias were excluded from the primary synthesis and reintroduced in a sensitivity analysis.

**Table 1:** PECOS framework.

Population	All age groups and diverse ethnic populations who work in a construction setting in West Africa
Exposure	Occupational health and safety in the construction industry including workplace hazards such as physical, chemical, ergonomic, musculoskeletal, psychological and biological hazards as well as occupational health and safety regulations, measures and compliance.
Comparator	Not applicable
Outcomes	General outcome: Analyze and synthesize the existing research on the compliance level of construction workers with occupational health and safety standards in West Africa. Specific outcomes: To identify factors associated with the compliance to occupational health and safety standards among construction workers in West Africa. To analyze reported strategies for improving compliance with occupational health and safety standards
Study design	Quantitative studies

### Operational definition and classification of outcomes

For this review, OHS compliance was operationally defined as adherence by workers to prescribed OHS practices or implementation by employers or sites of required OHS provisions. Outcomes were classified according to the construct actually measured in the primary study rather than treated as interchangeable indicators of a single compliance construct.

Worker-level behavioural compliance included reported or observed performance of prescribed safety practices, particularly PPE use and adherence to specified work procedures. Organisational and regulatory implementation indicators included employer PPE provision, safety policies, inspections, monitoring, accident reporting, safety-management arrangements, engineering or administrative controls, and welfare-facility provision. PPE access, PPE source/provision and PPE use were treated as distinct constructs. Safety knowledge, awareness and training were synthesized separately because they may support or be associated with compliance but do not themselves demonstrate behavioural compliance. Welfare-facility availability/provision was distinguished from adequacy or worker use where the primary study reported these separately.

### Search strategy

The original search covered PubMed, IEEE Xplore, EBSCOhost, ScienceDirect and Google Scholar through Harzing's Publish or Perish from June 2001 to 12 February 2025. The geographic search component combined ‘West Africa’ and regional descriptors with the individual names of all 16 West African countries. We used the following search terms: ‘Compliance’ and all applicable alternate terms crossed with ‘Construction workers’, ‘building workers’, and all applicable alternate terms crossed with the geographic search component of ‘West Africa’, ‘Ecowas region’, its applicable alternate terms including the individual names of all 16 West African countries. Boolean operators of “OR” and “AND” were used to increase the scope of studies eligible for screening. The search was restricted to English-language records. Complete database-specific search strings are provided in S2 Text.

The original three searches in Google Scholar retrieved 980, 829 and 509 records, respectively (2,318 in total). All records returned by the three searches were exported into the screening dataset; no arbitrary restriction to the first 100, 200 or another fixed number of ranked results was applied. At the Editor's request, the search was updated from 13 February 2025 through 18 August 2026 using the same information sources and eligibility criteria. The updated search retrieved 219 additional records: 12 from EBSCOhost, 1 from IEEE Xplore, 14 from PubMed, 55 from ScienceDirect and 137 from Google Scholar. The three updated Google Scholar searches returned 70, 50 and 17 records, and all were exported for screening.

### Selection process

The studies identified from the search were independently screened by two researchers via Rayyan Reference Manager. Each study had its title and abstract screened independently to obtain the relevant literature using the eligibility criteria. Studies adhering to the eligibility criteria were full-text reviewed by two independent researchers and the reasons for exclusion at this stage were documented. A record advanced when the screening decision supported further assessment. Differences in the screening results for both stages were resolved by a third independent reviewer. For studies whose full texts were not accessible through open-access sources or institutional subscriptions, additional database and library searches were performed.

### Quality assessment

The Joanna Briggs Institute (JBI) checklist for analytical cross-sectional studies was used to assess the risk of bias of the included cross-sectional studies [39]. Two independent reviewers assessed the risk of bias for each full-text article. Differences were resolved through a third independent reviewer. Studies rated as high risk of bias were excluded at the synthesis stage. The JBI items were not converted to a percentage score, and no numerical threshold was used to define overall risk of bias. Instead, the review team made domain-based overall judgments. Low risk indicated that the principal methodological domains were adequately addressed and remaining concerns were unlikely to materially affect the findings. Moderate risk indicated important limitations that were not considered sufficiently extensive to seriously undermine the findings, and high risk indicated multiple serious or unresolved concerns in key domains such as participant selection, exposure or outcome measurement, confounding or analytical validity. No single JBI item automatically resulted in exclusion. Seventeen studies met the eligibility criteria across the original and updated searches. Five were judged to have high overall risk of bias and were excluded from the primary synthesis, leaving 12 low- or moderate-risk studies. The registered protocol prespecified JBI appraisal and low/moderate/high categorisation but did not explicitly prespecify automatic exclusion of high-risk studies; exclusion from the primary synthesis was therefore treated as a protocol deviation. To assess the consequence of that decision, a sensitivity analysis reintroduced the five high-risk studies and compared the qualitative direction of findings with the primary 12-study synthesis. The quality assessment checklist for all studies is presented in S3 Text.

### Data extraction and synthesis

Data were extracted independently by two reviewers from all eligible full-text studies. The data extracted included the first author, year of publication, study country, study design, sample size, sample population characteristics (age, gender), compliance-related outcomes (PPE use, regulatory adherence, safety knowledge and training, and provision and use of welfare facilities), associated factors, and reported or proposed improvement strategies are summarized in Tables 2–4. Discrepancies were discussed and resolved by rechecking the primary source. During revision, a source-level audit was undertaken to verify denominators, distinguish access from provision and use, identify conditional percentages, and correct numerical or labeling inconsistencies in the submitted manuscript.

**Table 2:** Summary of the characteristics of the included studies.

Study ID	Author, Year	Study Designs	Study Country	Sample size	Population characteristics	
					Age	Gender (%)
Study 1 [42]	Agbede et al. (2016)	Cross-sectional	Nigeria	115	Not reported	Not reported
Study 2 [43]	Bonney (2016)	Cross-sectional	Ghana	306	<20-50	All genders Males = 271 Females = 35
Study 3 [44]	Rockson (2018)	Cross-sectional	Ghana	43	30+	Males
Study 4 [45]	Agyemang-Beniako (2019)	Cross-sectional	Ghana	110	20+	All genders Males = 93 Females = 17
Study 5 [46]	Hammond (2019)	Cross-sectional	Ghana	123	20-69	Males
Study 6 [47]	Afatsawu & Kheni (2022)	Cross-sectional	Ghana	201	Not reported	Not reported
Study 7 [48]	Boakye et al. (2022)	Cross-sectional	Ghana	173	18+	Males
Study 8 [49]	Friday & chika (2022)	Cross-sectional	Nigeria	463	18-55	All genders Males = 441 Females = 18 Unspecified = 4
Study 9 [50]	Tannor et al. (2023)	Cross-sectional	Ghana	201	All ages	All genders Males = 189 Females = 12
Study 10 [51]	Amadi & Udofia (2023)	Cross-sectional	Nigeria	480	20+	Not reported
Study 11 [40]	Ameen et al. (2026)	Cross-sectional	Nigeria	250	≥18	All genders Males = 230 Females = 20
Study 12 [52]	Moda et al. (2026)	Cross-sectional	Nigeria	560	18–50	All genders Males = 461 Females = 99

**Table 3:** Domain-specific compliance with OHS.

Study ID	Author, Year	PPE use/access /provision	Regulatory /organisational implementation	Knowledge/ awareness/ training	Welfare facilities /services
Study 1 [42]	Agbede et al. (2016)	Not reported	Policy implementation 50% Health and safety communication 62% Inspections 15% Accident records 10% Audits 20%.	Training programmes for site workers 25%	Sanitation/welfare facilities 39% (firm-level).
Study 2 [43]	Bonney (2016)	79.1% agreed that safety equipment is used	86% agreed safety is promoted at workplace 95.4% agreed with complying to OHS safety policies 87.2% agreed correct procedures are used	93.2% agreed workers pay attention to training and applied knowledge	Not reported
Study 3 [44]	Rockson (2018)	PPE provision selected as company practice: 27.9%	Worker involvement, communication and management practices reported.	Training selected as company practice: 11.6%	Welfare facilities selected as company practice: 9.3%
Study 4 [45]	Agyemang-Beniako (2019)	Not reported	Agree/strongly agree: daily monitoring 99%, Policies/guidelines 100%, Safety committee/representative 99.1%.	Hazard knowledge 100% Safety-sign knowledge 99.1% PPE-use knowledge 100%.	Not reported
Study 5 [46]	Hammond (2019)	PPE use: always 2.4%, occasionally 4.1%, when reminded 30.9%, when necessary 62.6% Selected availability: mask 11.4%, belt 0.8%, gloves 16.3%, goggles 22.8%, earplugs 0.8%.	Study-reported overall OHS compliance 73.2% in Results (abstract 72.4%)	Overall awareness 87.8%, policy awareness 98.4%, PPE-protocol awareness 99.2%; extinguisher training 75.6%.	Not reported
Study 6 [47]	Afatsawu & Kheni (2022)	Nose-mask use when exposed/likely exposed to hazardous chemicals: 58%.	Factory Inspectorate inspection 78%, accident notification 72%, FOS Act compliance 72%, registration 71%; PPE provision requirement 53%, firefighting equipment 67%.	67.7% agreed employer provided training or education	Not reported
Study 7 [48]	Boakye et al. (2022)	Access: helmet 92.5%, earplug/muff 5.8%, harness/belt 11.5%, mask 23.1%, boots/shoes 96.0%, gloves 37.6%, vest 89.6%, goggles 12.7%.	Not reported	Approximately nine in ten respondents reported PPE-use instruction/training in the source	Not reported
Study 8 [49]	Friday & chika (2022)	Protective materials 40.6%, hard hat 34.1%, foot protection 55.3%, goggles while inspecting 13.4%, mask while mixing/demolition 37.3%, mask while sanding/drilling 28.1%, respirator 1.7%, fall harness 28.1%	Not reported	Good knowledge 70.2% Training source: company 44.1%, self 30.2%, roadside 24.8%.	Not reported
Study 9 [50]	Tannor et al. (2023)	Helmet provision 44.3% / observed use 16.9%, gloves provision 19.9% / use 8%, goggles provision 2% / use 0%, footwear provision 16.4%, while all observed workers wore footwear but protective features were limited.	Site-level PPE provision and welfare implementation observed.	Not reported	Flush toilet 14.3%, wash basin 2.9%, washing facilities 2.9%, changing room 2.9%, dining area 22.9%, urinal 94.3%
Study 10 [51]	Amadi & Udofia (2023)	PPE available to 66.7%	Study-reported good safety practice 61.9%.	Safety training 42.7%, good safety practice 78.0% among trained vs 49.8% among untrained.	Not reported
Study 11 [40]	Ameen et al. (2026)	PPE classified as regular vs inconsistent; internal category-count inconsistency prevented derivation of whole-sample prevalence.	Not reported	No-safety-training associated with injury (AOR 2.47, 95% CI 1.42-4.32); training prevalence not derived from internally inconsistent category counts.	Not reported
Study 12 [52]	Moda et al. (2026)	54.5% did not wear a mask, 23.8% always wore one, 37.9% used cloth/handkerchief, 37.7% reported no PPE for dust exposure	Water suppression always 15.0%, design/alternative dust control always 17.5%, regular health checks always 20.5%, 66.1% perceived very little dust-risk-control effort.	55.0% completely unaware of dust-related health risks, 15.2% fully aware, 24.3% fully aware of safety measures for dust-generating tasks.	Not reported

**Table 4:** Factors associated with OHS compliance and Reported strategies to improve OHS.

Study ID	Author, Year	Factors associated with OHS compliance	Reported Strategies to improve OHS
Study 1 [42]	Agbede et al. (2016)	Rewarding workers for safe site behaviour Bureaucracy Cost Poor enforcement and awareness Low involvement of top management in health and safety	Informing employees about hazards on sites before work starts Designing site rules and measures to mitigate assessed risks Providing a designated health and safety department Engaging workers on health and safety issues Effective display and communication of health and safety information Health and safety for site supervisors/managers Provision of training programmes and awareness raising initiatives Tightening of health and safety regulation
Study 2 [43]	Bonney (2016)	Poor communication on sites Time pressures Lack of safety rules & guidelines Poor safety supervision by site managers/foremen Too much workload Lack of proper safety education & training Lack of PPE Lack of management commitment Complicated safety rules & regulations Non-enforcement of safety rules & regulations Overconfidence in taking risk/not being risk averse	Site induction and refresher training Proper communications and interpersonal skills Management should deliver effective safety management systems
Study 3 [44]	Rockson (2018)	Worker involvement in health and safety decisions Health and safety awareness and knowledge	Involvement of workers in health and safety decisions Daily briefing of health and safety issues Workshops & Refresher courses on health and safety Incentives to workers who are committed to complying with health and safety standards
Study 4 [45]	Agyemang-Beniako (2019)	Not reported	Provision of appropriate protective equipment and clothing Effective and regular safety training Consultants should identify risks and hazards that can cause accidents in the construction site and minimize them during the construction project Comprehensive legislation of OHS policy should be implemented by Government of Ghana Establishment of an OSHA department for inducting punishment for safety violators
Study 5 [46]	Hammond (2019)	Not reported	Enhancing promotion of OHS practices and awareness by employers Intensifying training and supervision of PPEs by employers Employing managers with relevant capabilities to ensure high OHS compliance
Study 6 [47]	Afatsawu & Kheni (2022)	Not reported	Construction companies should stimulate self-compliance to health and safety regulations Enforcement of health and safety standards by construction firms Institute training of workers in regards to OHS compliance
Study 7 [48]	Boakye et al. (2022)	Feeling too hot when using PPEs Difficulty in doing tasks when using PPEs To save time Poor vision when using PPEs PPEs tends to fall off	On the job training with practical demonstrations
Study 8 [49]	Friday & chika (2022)	Educational background: primary (0%), secondary (24%), tertiary (70.4%), Non-formal (0%) Safety training: Self (8.6%), Roadside (7%), Company (58.8%) Age: 18–25 (0%), 26–35 (39.1%), 36-45 (23.3%), 46-55 (59.8%) Years of experience: 1–5 (7.7%), 5-10 (58.5%), 11-15 (17%), 16+ (44.4%)	Award incentives to workers who comply with OHS Enforcement of OHS regulations by policy makers and law enforcement agencies Periodic safety education by safety officers on construction sites
Study 9 [50]	Tannor et al. (2023)	Lack of regulatory framework in the Ghanaian construction industry	Implement and enforce use of welfare facilities and PPEs by construction firms
Study 10 [51]	Amadi & Udofia (2023)	Educational background: primary (17.4%), secondary (82.1%), tertiary (21.8%) Safety training: Trained (78%), No training (49.8%) Age: 20–30 (68.1%), 31–40 (60.6%), 41-50 (45.8%), 51+ (80%) Years of experience: 1–5 (59.2%), 6-10 (75.8%), 11-15 (19.1%)	Provision and enforcement of use of PPEs by companies Organization of safety training programmes by safety training bodies
Study 11 [40]	Ameen et al. (2026)	Lack of training	Mandatory or periodic training Consistent PPE usage Supervision and routine inspection
Study 12 [52]	Moda et al. (2026)	Age Education Work schedule	Tailored education Worker engagement and health checks

Meta-analysis was considered inappropriate because outcome definitions, measurement instruments, PPE items, denominators, units of analysis and statistical reporting differed substantially across studies. A structured narrative synthesis was therefore conducted around four predefined OHS compliance-related domains: (1) PPE use, access and provision; (2) regulatory and organisational implementation; (3) safety knowledge, awareness and training; and (4) welfare-facility provision and use.

For each domain, findings from all studies reporting relevant data were tabulated and synthesized collectively. Study-specific percentages, proportions, means and association measures were retained in the form reported by the primary study. Where studies measured sufficiently comparable constructs, ranges were described, outcomes representing different constructs or substantially different denominators were not combined. No review-generated composite compliance score was calculated. Original study-reported overall or domain-specific scores were retained only when clearly identified as study-reported measures. Missing or unreported data were not imputed. Where a denominator or outcome definition could not be reliably established, the value was either reported with an explanatory note or omitted from comparative summaries.

Factors associated with compliance-related outcomes were grouped as individual, work-related, organisational and regulatory/system-level factors. Strategies were summarized only as reported or proposed by the primary studies because no included study evaluated an intervention. Causal terminology was avoided throughout because all included studies were cross-sectional

### Sensitivity analyses

Two narrative sensitivity analyses were undertaken. First, publication status was examined by comparing the primary 12-study synthesis with the seven peer-reviewed studies alone; the remaining five non-peer-reviewed studies comprised four postgraduate theses/dissertations and one conference proceeding. Publication status was not treated as a proxy for methodological quality because JBI risk of bias was assessed separately. Second, the risk-of-bias sensitivity analysis compared the 12-study primary synthesis with all 17 eligible studies after reintroducing the five studies rated at high risk. No pooled or composite estimate was calculated in either sensitivity analysis.

### Protocol deviations

The registered protocol additionally proposed examining the impact of OHS non-compliance on worker health and safety. Because the evidence base was entirely cross-sectional, the revised review does not interpret observed associations as causal impact. One study identified in the search examined cross-sectional associations between safety training, PPE use and occupational injury, but this does not establish temporal or causal effects [40]. The revised synthesis therefore focuses on compliance-related outcomes, factors associated with those outcomes, and strategies reported or proposed for improvement. As noted above, exclusion of high-risk studies from the primary synthesis was also not explicitly prespecified and was addressed transparently through sensitivity analysis.

## Results

### Study selection

The results are presented in a flow diagram according to the PRISMA guidelines (Fig 1). The literature search revealed a total of 2724 studies. After removing 759 duplicates, 1965 studies underwent title and abstract screening. Following the title and abstract screening, 1718 studies were excluded leaving 247 studies for eligibility assessment. Among the 247 studies for eligibility assessment, 230 studies were excluded after full text review. Of the 230 excluded studies, 168 did not meet our PECOS criteria as they did not address actual compliance with occupational health and safety among construction workers in West Africa, 36 studies had their full text being inaccessible, 10 of the studies were conference papers without separable quantitative data and 8 studies were book chapters and 8 studies also had their compliance data not extractable. 17 studies met the inclusion criteria but 5 studies were excluded because of their high risk of bias. Common reasons for their exclusion included unclear inclusion criteria, non-validated outcome measures and absence of confounder handling. Ultimately, 12 studies were selected for the systematic review.

**Fig 1:**
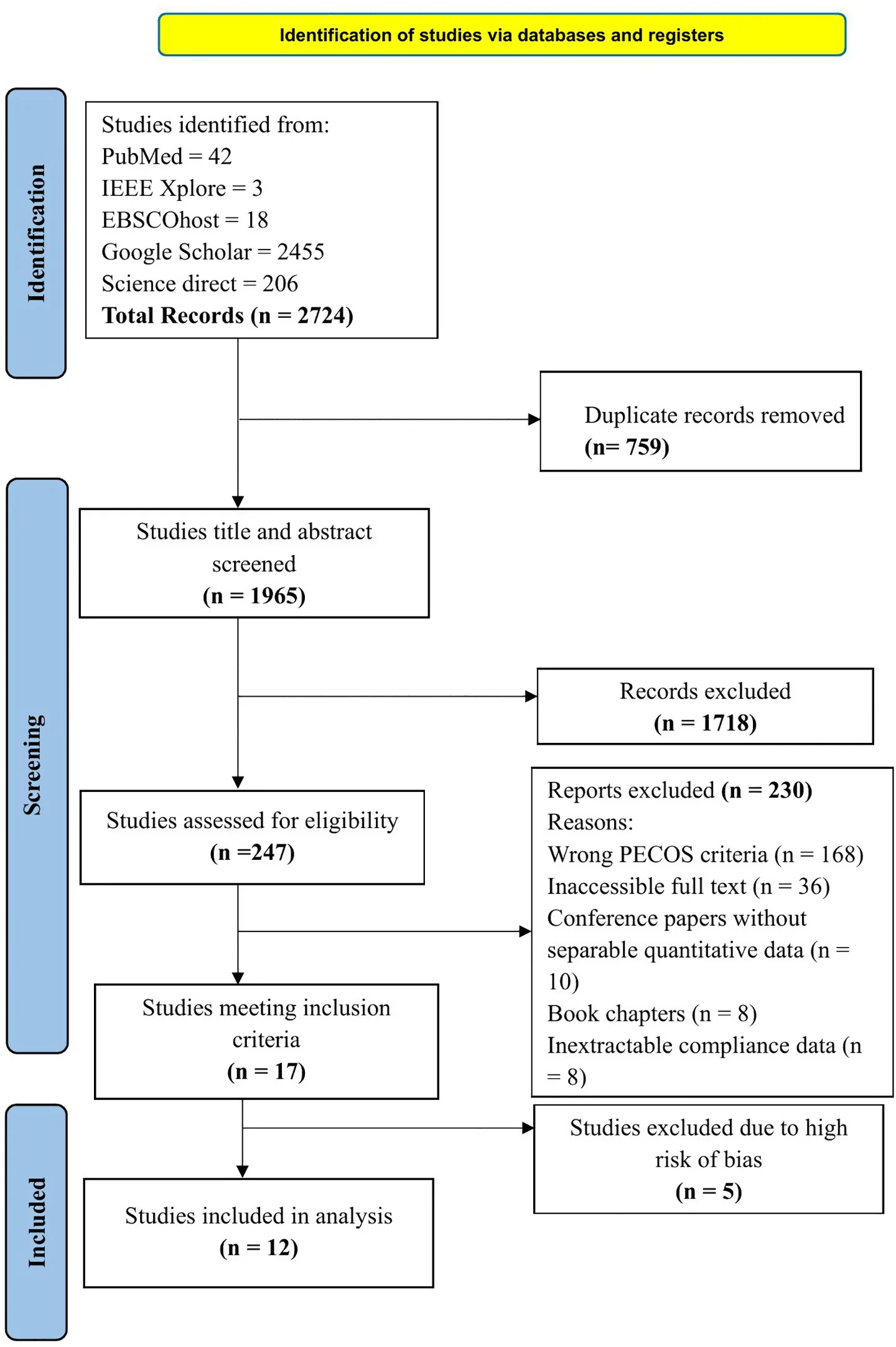
Flow diagram of study selection.

### Study characteristics

All 12 cross-sectional studies were conducted in Ghana (n = 7) and Nigeria (n = 5), published between 2016 and 2026, reflecting a recent evidence base. Using this review’s search sources and eligibility criteria, no eligible low‑ or moderate‑risk studies were identified from the other 14 West African countries. The evidence base comprised peer-reviewed articles (n = 7) and non-peer-reviewed sources (n = 4 postgraduate theses or dissertations and n = 1 conference proceeding). The sample sizes ranged from 43 to 560 participants. Tannor et al. additionally included observation of 35 construction sites. Because some studies used site-level observations, conditional denominators or multiple levels of analysis, a single summed participant denominator was not used as a review-level estimate. The study populations were predominantly male, reflecting the demographic structure of the construction sector in the region. Most studies focused on building construction workers, artisans, carpenters, and labourers. Rockson (2018) studied workers employed by D1K1- and D2K2-classified contractors in Ghana. Under Ghana's contractor classification system, D denotes building works and K denotes civil works, while the numerical class denotes contractor capacity, with Class 1 representing the highest classification [41]. The characteristics of the included studies are summarized in Table 2.

### Quantitative evidence on OHS compliance-related outcomes

#### PPE use, access and provision.

PPE was the most frequently reported OHS domain, but direct comparison was limited by differences between use, access, provision and task-specific practice (Table 3). Hammond reported that 2.4% of workers said they always used PPE, 4.1% occasionally, 30.9% when reminded and 62.6% when they considered it necessary. Tannor et al. observed helmet use in 16.9% of workers, glove use in 8.0% and goggle use in 0%, while Friday and Chika reported hard-hat use in 34.1%, foot protection in 55.3%, fall-arrest harness use in 28.1%, respirator use in 1.7%, and task-specific mask use of 28.1% during sanding/drilling and 37.3% during mixing/demolition.

Access and use were not treated as equivalent. In Boakye et al., access was high for helmets (92.5%), safety boots/shoes (96.0%) and safety vests (89.6%) but low for earplugs/muffs (5.8%), safety harnesses/belts (11.5%) and goggles (12.7%). Frequency-of-use percentages in that study were conditional on having access to the specific PPE item. For example, among 19 workers with access to a harness/belt, 42.1% reported always using it. Among 166 with access to safety boots/shoes, 59.0% reported always and 6.6% often using them.

The Nigerian study by Moda et al. further illustrated respiratory-protection gaps: 54.5% reported not wearing a mask, 23.8% reported always wearing one, 37.9% used cloths or handkerchiefs and 37.7% reported using no PPE for dust exposure. Ameen et al. classified PPE use as regular versus inconsistent but contained category counts that were not arithmetically compatible with the total sample. Consequently, no whole-sample PPE prevalence was derived from those counts. Its adjusted association between inconsistent PPE use and injury was retained in the factors synthesis.

#### Regulatory and organisational implementation.

Organisational and regulatory indicators also varied in construct and level of analysis (Table 3). In the Agbede firm survey, 50% reported an implemented health and safety policy, 62% a health and safety communication arrangement, 25% training programmes for site workers, 15% regular site inspections, 39% sanitation/welfare facilities, 10% accident records and 20% safety audits. Afatsawu and Kheni reported respondent-reported regular Factory Inspectorate inspection (78%), accident notification (72%), compliance with provisions of the Factories, Offices and Shops Act (72%), registration with the Factory Inspectorate (71%), Labour Act-related PPE provision (53%) and installed firefighting equipment (67%). These are organisational/regulatory implementation indicators rather than direct worker-behaviour measures.

Agyemang-Beniako reported very high agreement with statements on daily monitoring/inspection (99%), organisational policies/guidelines (100%) and active safety committees/representatives (99.1%); these were respondent perceptions and were not interpreted as independently observed inspection rates. In Moda et al., only 15.0% reported that water suppression was always provided, 17.5% reported dust control through design/alternative methods was always used, and 20.5% reported regular health checks were always provided; 66.1% perceived very little industry effort in dust-risk control.

#### Safety knowledge, awareness and training.

Knowledge, awareness and training were synthesized separately from behavioural compliance. Several studies reported high general OHS knowledge or awareness while specific protective practices remained variable (Table 3). Hammond reported 87.8% overall awareness of OHS protocols, and Agyemang-Beniako reported near-universal agreement with several knowledge statements. Friday and Chika reported 70.2% good knowledge; the reported source of safety training was company-based for 44.1%, self-training for 30.2% and informal roadside instruction for 24.8%.

Amadi and Udofia reported that 205 of 480 workers (42.7%) had received safety training, and good safety practice was more common among trained than untrained workers (78.0% versus 49.8%). Evidence from Moda et al. added a different pattern: It found that 55.0% were completely unaware of dust-related health risks and only 15.2% were fully aware; 24.3% were fully aware of safety measures for dust-generating tasks. Ameen et al. found that lack of safety training was associated with occupational injury after adjustment (AOR 2.47, 95% CI 1.42-4.32), but the cross-sectional design precludes causal interpretation.

#### Provision and use of welfare facilities and services.

Welfare-facility evidence was less frequently reported (Table 3). Agbede reported sanitation and welfare facilities in 39% of surveyed firms. Rockson identified welfare provision as one of several company practices selected by 9.3% of respondents; because that value arose from a company-practice choice question, it was not interpreted as a site prevalence. Tannor et al. provided the most detailed site-level evidence: among 35 sites, 14.3% had flushable toilets, 2.9% had wash-hand basins/sinks, 2.9% had washing facilities, 2.9% had changing rooms and 22.9% had dining areas. The study also reported very high urinal availability and substantial drinking-water provision, but some welfare items contained internal differences between narrative and tabular reporting; only clearly interpretable items were used in comparative summaries.

### Factors associated with OHS compliance-related outcomes

Associated factors were grouped into individual, work-related and organisational or regulatory levels (Table 4). At the individual level, Friday and Chika reported associations of age and educational attainment with occupational-hazard knowledge, while Amadi and Udofia reported associations of age, education, training, work experience and PPE availability with study-defined safety practice. Moda et al. found that age, educational qualification, work schedule, region and smoking status were associated with selected silica-dust awareness and practice measures. These cross-sectional associations do not establish temporal direction.

At the work-related level, Boakye et al. found that employment type was associated with perceived barriers and motivating factors for PPE adherence. Reported reasons for removing PPE included heat, task interference, poor vision and time pressure. Ameen et al. found that no safety training (AOR 2.47, 95% CI 1.42-4.32), inconsistent PPE use (AOR 1.76, 95% CI 1.03-3.01) and more than five years of work experience (AOR 1.52, 95% CI 1.01-2.66) were associated with occupational injury. Age 40 years or older was not statistically significant in that adjusted model.

At organisational and regulatory levels, included studies repeatedly reported or proposed roles for PPE availability, supervision, communication, worker involvement, inspection and regulatory enforcement.

### Reported strategies to improve compliance with OHS

No included study tested the effectiveness of an OHS intervention. The evidence therefore supports only a summary of strategies reported or proposed by primary-study authors. Recurring proposals included practical and refresher safety training, reliable and appropriate PPE provision, enforcement of PPE use, routine inspection and monitoring, worker involvement in safety decisions, toolbox talks and hazard communication, management commitment, safety incentives, improved record keeping, engineering or administrative dust controls, adequate welfare facilities, and stronger construction-specific OHS governance (Table 4). These approaches should be interpreted as priorities for evaluation rather than demonstrated effective interventions.

### Cross-country patterns

The available Ghanaian and Nigerian evidence differed in emphasis rather than demonstrating a clear country-level ranking. Ghanaian studies more often reported site-level implementation, PPE access or provision, inspections and welfare facilities, whereas several Nigerian studies focused on worker knowledge, practice, training, dust-control behaviour and occupational injury. These differences may reflect the specific questions, populations and measurement tools selected by researchers rather than true national differences. The review therefore does not infer that one country has better OHS compliance than the other.

### Sensitivity analyses

#### Publication-status sensitivity.

The primary synthesis included 12 studies: seven peer-reviewed articles and five non-peer-reviewed sources. Restricting the synthesis to the seven peer-reviewed studies did not materially change the qualitative direction of the main findings. The peer-reviewed evidence alone still showed wide variability in PPE use and access, low use of several specialised PPE items, inconsistent organisational implementation, marked variation between knowledge or awareness and reported practices, and deficits in selected welfare facilities. The non-peer-reviewed studies broadened the evidence on worker perceptions and organisational practices but did not reverse these patterns. Publication status was not interpreted as a quality ranking because JBI risk of bias was assessed independently

#### Risk of bias sensitivity.

Reintroducing the five high-risk studies increased the breadth and heterogeneity of the evidence but did not materially reverse the principal domain-level interpretation. The additional studies contributed further examples of low or inconsistent PPE practices, training and implementation gaps, but their methodological limitations reduced confidence in the precision of those estimates. The primary conclusions were therefore not dependent on exclusion of the high-risk studies.

## Discussion

### Summary of principal findings

This review identified 17 eligible cross-sectional studies and retained 12 low- or moderate-risk studies in the primary synthesis. The evidence was geographically concentrated in Ghana and Nigeria despite a West Africa-wide search. Across the four predefined OHS compliance-related domains, the principal finding was heterogeneity rather than a defensible single ‘overall compliance rate.’ PPE access, provision and use differed markedly by equipment type, organisational and regulatory implementation indicators ranged from infrequently reported practices to high respondent agreement with selected policies, knowledge and awareness were sometimes high while protective practices remained variable, and detailed site-level welfare evidence showed important deficits in basic facilities. The updated search added two Nigerian studies and strengthened rather than reversed these patterns.

The revised synthesis also changes how the evidence should be interpreted. Access to PPE is not the same as employer provision, and neither is equivalent to use. Knowledge of a safety requirement is not behavioural compliance. Similarly, respondent agreement that inspections occur is not equivalent to an independently observed inspection rate. Removing review-generated composite scores and retaining study-specific constructs therefore reduces apparent comparability but produces a more valid representation of the evidence

### Comparison with global literature

When situated within the global literature, these findings reflect broader patterns observed in low- and middle-income countries (LMICs). Studies in Ethiopia and Bangladesh report difficulty with PPE use and regulatory adherence [53,54]. What distinguishes the present review is its domain-specific synthesis of the Ghanaian and Nigerian evidence, which reveals that compliance is not only low but also fragmented. This fragmentation suggests that interventions targeting single domains (e.g., PPE provision) may be insufficient unless embedded within broader system-level reforms.

In contrast, high-income countries with dedicated construction OHS frameworks and proactive inspection regimes have achieved significantly lower injury rates [55]. The United Kingdom’s Health and Safety Executive, for example, conducts routine proactive inspections with meaningful penalties for violations, contributing to sustained compliance improvements [56]

### The knowledge-practice gap

One of the strongest findings of this review is the persistent disconnect between safety knowledge, and actual compliance behaviour. High levels of reported awareness coexisted with low use or access for selected PPE items. PPE-protocol awareness and overall awareness of OHS protocol was very high, yet compliance behaviour was low: PPE-protocol awareness and overall OHS protocol awareness was 99.2% and 87.8% respectively, yet PPE use was 2.4% for always, 4.1% for occasionally, 30.9% when reminded and 62.6% when necessary [46]. This finding challenges the dominant assumption in many OHS interventions that increasing knowledge directly translates into safer practices [57]. Behavioural science models such as the Health Belief Model and Theory of Planned Behavior emphasise that behaviour is shaped by perceived susceptibility, barriers, social norms, and self-efficacy, not knowledge alone [58,59].

The magnitude of the gap observed in this review suggests several explanations. First, the content and quality of training matters. Workers who received company-based training (44.1%) demonstrated better knowledge than those who were self-trained (30.2%) or learned through informal “roadside” instruction (24.8%), indicating that training quality matters as much as training quantity [49]. Second, physical barriers override knowledge. 90.9% of workers removed safety boots because they felt too hot, 68.5% removed safety harnesses to save time, and 40.9% removed goggles due to poor vision [48]. These are not excuses but genuine constraints in a hot, humid, labour-intensive environment where productivity is directly linked to income. Third, economic pressures incentivize speed over safety, particularly for casual workers. These findings suggest that training-only interventions are unlikely to produce sustained behavioural change, but rather should be combined with reliable PPE provision, worker-centred selection, supportive supervision, enforcement, and protection for casual workers.

### Welfare facilities: The neglected domain

The neglect of welfare facilities documented in this review is both striking and consequential. Welfare facilities were among the weakest compliance domains. The finding that drinking water was provided on 100% of sites while flushable toilets (14.3%), washing facilities (2.9%), and changing rooms (2.9%) were almost entirely absent suggests that contractors prioritise the least costly and most visible welfare provision. This neglect has direct implications for worker health: Lack of access to toilets and washing facilities encourages workers to delay urination and defecation, which can lead to urinary tract infections, constipation, and other health problems [60]. This neglect can also increase risk of gastrointestinal infection from an inability to wash hands before eating, and exposure to hazardous materials brought home on contaminated clothing due to a lack of changing rooms. The near-complete absence of welfare facilities also reflects a broader devaluation of construction workers’ humanity. The message sent by an employer who provides no toilet, no place to wash, and no place to eat is clear: the worker's basic needs do not matter. This devaluation is likely to affect worker morale, job satisfaction, and willingness to comply with other safety requirements [61]. Improving welfare facilities may have spillover effects on other compliance domains by signalling that the employer values worker well-being.

### The regulatory void

Lack of comprehensive OHS policy, poor infrastructure and funding, insufficient number of qualified occupational health and safety practitioners, and the general lack of adequate information are among the main drawbacks to the provision of effective enforcement and inspection services in most African countries [62]. This was supported by Tannor et al. (2023) which explicitly identified the lack of a dedicated regulatory framework for occupational health and safety in Ghana's construction industry as the root cause of non-compliance. Ghana has not ratified ILO Convention 155, and there is no single, harmonised OHS law specifically designed for the construction sector. Instead, construction firms are covered by fragmented legislation (Labour Act 2003, Factories Offices and Shops Act 1970) that was not designed for construction-specific hazards. Ghana's first national OHS Profile, launched in 2025, identified policy gaps, institutional weaknesses, resource constraints, inspector shortages and risks associated with the informal economy, and was intended to support development of a more coordinated national OSH framework [63]. This regulatory void means there are no legally binding standards for welfare facilities, no mandated ratios of safety officers to workers, no requirements for site-specific safety plans, and no systematic accident reporting or investigation. Ghana has however ratified 10 conventions out of over 70 conventions of the ILO that are OHS related [38]. However, the four core conventions on OHS have all not been ratified [62].

Nigeria faces similar challenges, having only recently ratified ILO Convention 187 [64]. Nigeria has the Factories Act 2004 and a Revised National Policy on Occupational Safety and Health 2020 that applies across formal and informal workplaces; the Federal Ministry of Labour and Employment's OSH Department reports inspection, investigation, audit and enforcement functions under this framework [65].

### Geographic and evidence gaps

No eligible low- or moderate‑risk studies were identified from 14West African countries. This should not be interpreted as evidence that no relevant or methodologically robust research exists in those countries. The English-language restriction and the omission of Scopus, Web of Science and Embase may have reduced retrieval of studies, particularly from Francophone and Lusophone countries. The finding therefore identifies a gap in the present review’s eligible base, not an established absence of country-level OHS research or policy evidence.

### Limitations of the evidence base

The findings of this systematic review must be interpreted in light of several limitations of the underlying evidence base. First, all included studies were cross-sectional, precluding causal inference. Second, many studies relied on respondent reports which may be affected by recall or social desirability bias, although some studies also reported direct worker or site observations. Third, the definition and measurement of compliance varied substantially across studies, limiting comparability. Fourth, the evidence base was limited to Ghana and Nigeria, with no data from other West African countries. The English-language restriction may have introduced geographic selection bias by reducing sensitivity to evidence from Francophone and Lusophone West Africa. Scopus, Web of Science and Embase were not searched, which may have resulted in missed eligible studies despite the use of multiple bibliographic sources and an extensive Google Scholar search. Finally, inclusion of non-peer-reviewed studies broadens the evidence base but may increase variability in reporting quality; this concern was addressed through separate JBI appraisal and publication-status sensitivity analysis.

### Strengths of this review

This is the most comprehensive systematic review of OHS compliance among construction workers in West Africa to date, synthesising 12 cross-sectional studies as having low or moderate risk of bias. The review followed PRISMA guidelines, was registered with PROSPERO, used the JBI critical appraisal tool to exclude high‑risk studies, and expanded beyond peer-reviewed literature to include eligible grey literature, which is important in African construction-safety research. The narrative thematic synthesis preserved the complexity of compliance by separating domains rather than forcing heterogeneous studies into a single pooled estimate.

## Conclusion

Available quantitative evidence from Ghana and Nigeria indicates substantial variability and important gaps across OHS compliance-related outcomes among construction workers. PPE use and access were uneven across equipment types, organisational and regulatory implementation varied by setting and measure, safety knowledge and training did not consistently correspond with protective practices, and selected welfare facilities were inadequately provided. One cross-sectional study found that lack of safety training and inconsistent PPE use were associated with occupational injury, but causal relationships cannot be established. Because the West Africa-wide search yielded eligible primary-synthesis evidence only from Ghana and Nigeria, regional generalizability remains limited. Prospective multicountry studies using standardized outcome definitions and intervention studies evaluating proposed OHS strategies are needed to strengthen the evidence base

### Research priorities

Several priorities for future research emerge from this review. First, comparable multi-country studies should use standardized definitions, measures and transparent denominators to improve geographic coverage. Second, longitudinal studies and reliable administrative surveillance systems could help clarify temporal relationships and better quantify health and economic impacts (injury rates, disease burden, mortality, lost productivity). Third, intervention studies are needed to test the effectiveness of specific strategies for improving compliance, including training programmes, enforcement approaches, economic incentives, worker participation mechanisms, and industry-led voluntary initiatives.

### Recommendations for policy and practice

Despite the evidence gaps, the findings of this review support several actionable recommendations for policymakers, employers, worker organisations, and researchers.

For policymakers, the development and enforcement of a dedicated construction specific OHS framework should be a priority. Such a framework must include legally binding standards for welfare facilities, PPE provision, safety training, and accident reporting, accompanied by adequate resourcing of labour inspectorates and meaningful penalties for violations.

For employers, investing in lightweight, breathable PPE designed for tropical climates, providing accessible and hygienic welfare facilities, and implementing targeted safety training for young and casual workers can improve compliance while potentially improving productivity and worker morale.

For worker organisations, advocating for the rights of casual workers, establishing peer safety committees, and providing anonymous reporting mechanisms can give workers a voice in safety decisions.

For researchers, building local research capacity, establishing regional research collaborations, and conducting intervention studies that test specific strategies for improving compliance can generate the evidence needed to guide policy and practice.

The construction industry is essential to West Africa's development but development that comes at the cost of workers’ health and safety is not sustainable. Ensuring that construction workers can do their jobs without sacrificing their health, their safety, or their dignity is not only a legal obligation but a moral imperative. The evidence reviewed here suggests that West Africa specifically Ghana and Nigeria are far from meeting the imperative of safe work, but the evidence also points the way forward: targeted interventions, enforceable standards, and a commitment to worker well-being can transform construction from one of the most dangerous occupations to one of the safest. The question is not whether this transformation is possible, but whether there is the will to make it happen.

## Supporting information

S1 Text Registered protocol for the systematic review. (DOCX)

S2 Text Full database-specific search strategies employed in this systematic review. (DOCX)

S3 Text JBI risk of bias assessments for peer-reviewed and non-peer-reviewed studies. (DOCX)

S1 Data Study-screening records, full-text exclusion reasons, eligibility decisions, extracted data and risk of bias assessments. (XLSX)

S1 PRISMA Checklist PRISMA 2020 checklist with manuscript page references. (DOCX)

## Abbreviations

OHS: Occupational health and safety
PPE: Personal protective equipment
PRISMA: Preferred Reporting Items for Systematic Reviews and Meta-analyses
PROSPERO: International Prospective Register of Systematic Reviews
PECOS: Population, exposure, comparator, outcome and study design
JBI: Joanna Briggs Institute
ILO: International Labour Organization
LMICs: Low- and Middle-Income Countries
AOR: Adjusted odds ratio
CI: Confidence interval
