# Supplementary material for: Occupational health and safety standards compliance among construction workers in West Africa: A systematic review of available evidence from Ghana and Nigeria
Source: PLOS Glob Public Health. 2026 Nov 13;6(11):e0006724. doi: 10.1371/journal.pgph.0006724 (PMC13766276; doi:10.1371/journal.pgph.0006724)
Supplement: Supplementary file 1 [file pgph.0006724.s006.pdf]

Reviewer’s Report

Manuscript Number: PGPH-D-26-02159

Title: Occupational health and safety standards compliance among construction workers in Ghana: A systematic review evidence from Ghana and Nigeria

General Evaluation

This manuscript addresses a critical occupational health gap within the West African construction sector, specifically synthesizing compliance with occupational health and safety (OHS) standards in Ghana and Nigeria. The study highlights important structural and behavioral issues, such as the knowledge-practice gap and the impact of precarious employment conditions on worker safety. However, several major methodological inconsistencies, severe data mismatches between the narrative text and tables, and geographical scope presentation issues must be resolved before this manuscript can be considered for publication.

### Major Concerns

1. Title Inconsistency and Scope Definition

Issue: There is a logical contradiction and redundancy in the current title: "Occupational health and safety standards compliance among construction workers in Ghana: A systematic review evidence from Ghana and Nigeria." The primary title restricts the geographic focus strictly to Ghana, which directly contradicts the methodology and inclusion criteria that encompass the wider West African region (specifically extracting data from both Ghana and Nigeria).

Recommendation: The title should be streamlined to reflect the actual regional scope defined in the methodology without adding redundant country clauses. The authors are recommended to revise the title to something more concise and professional, such as:

1: "Occupational health and safety standards compliance among construction workers in West Africa: A systematic review"

2: "Compliance with occupational health and safety standards among West African construction workers: A systematic review"

2. Weak Justification of the Knowledge Gap and Rationale in the Introduction

Issue: The Introduction currently provides a broad overview of the construction industry and OHS general statistics, but it fails to articulate a compelling "Knowledge Gap." It remains unclear whether previous systematic reviews on OHS compliance in developing countries or Africa exist, and if so, what specific limitations they possess that this study aims to rectify. Furthermore, the selection of the "West African" regional scope is introduced without a clear underlying rationale or contextual justification (e.g., shared regulatory frameworks, economic ties, or common labor market characteristics).

Recommendation: The authors need to heavily strengthen the Introduction by addressing the following points:

1. Explicitly state what is uniquely missing from current global or regional OHS literature regarding construction workers.

2. Provide a clear rationale for choosing West Africa as the defined scope. Explain the socio-economic or regulatory commonalities of this region that justify examining them under a unified systematic review.

3. Conclude the Introduction with a clear statement regarding how this synthesis will translate into global public health actions or regional policy-making, aligning better with the scope of *PLOS Global Public Health*.

3. Incomplete Description of the Study Selection and Conflict Resolution Process in Methods

Issue: In the Methods section, the authors state that two independent reviewers conducted the screening and selection of articles. While utilizing two reviewers meets the minimum standard for a systematic review, the manuscript completely lacks a detailed explanation of the "Conflict Resolution Process." It is not specified how disagreements between the two primary reviewers regarding study eligibility or quality assessment were resolved.

4. Potential Missing Studies & Search Strategy Limits

Issue: The PECOS framework defines the targeted population as *"all age groups... who work in a construction setting in West Africa"*. The authors note that no eligible studies were identified from the other 14 West African nations. However, it is unclear if individual country names (e.g., Liberia, Sierra Leone, Senegal) were explicitly included in the search strings, or if only regional umbrella terms ("West Africa", "ECOWAS") were used.

Recommendation: Please display the precise search string used for at least one major database (e.g., PubMed) in the main text or appendix to prove the sensitivity of the search. Additionally, since many West African nations do not use English as their official language (e.g., countries using French or Portuguese), the strict English-language restriction should be explicitly addressed in the Discussion as a limitation regarding potential geographic selection bias.

5. Vague and Unstructured Data Synthesis and Statistical Analysis Section

Issue: The "Data synthesis and statistical analysis" section is currently very superficial and lacks operational detail. While the authors state that a "narrative synthesis" was conducted due to the heterogeneity of the data, they fail to specify the structured framework used to guide this synthesis (e.g., whether the synthesis was structured by compliance domains, geographic locations, or study designs). Merely summarizing the findings of individual studies consecutively does not constitute a rigorous systematic narrative synthesis.

6. Severe Inconsistencies and Mismatches Between Text and Tables

Issue: There are multiple conflicting statistical values and reporting discrepancies between the text and the corresponding data tables. For example:

1. **Page 16 (Lines 269–271):** The text states, *"Boakye et al. (2022) reported that **70%** of Ghanaian workers used earplugs."* However, looking at **Table 4 (Page 22)** under the Boakye et al. (2022) row, it reports: *"Access to PPEs: ... Earplug (**5.8%**)"*. Access cannot logically be lower than actual usage.

2. **Page 40 (Line 335):** The narrative points out that welfare facilities were among only **9.3%** of practices in *Rockson (2018)*. Yet, in **Table 4 (Page 21)**, the entry for Rockson (2018) claims: *"Provision of PPE 27.9%"*.

Recommendation: The authors must perform a rigorous data audit across the entire manuscript to ensure that every percentage, sample size, and finding cited in the narrative exactly mirrors the data extracted into Tables 2 through 7.

7. Evidence Quality Weighting (Grey Literature vs. Peer-Reviewed)

Issue: The review includes 5 peer-reviewed journal articles and 5 postgraduate theses/dissertations. While including grey literature is excellent for mitigating publication bias, treating unpublished academic theses with the identical weight as peer-reviewed publications without adjusting the narrative synthesis can compromise robustness.

Recommendation: Please add a brief sensitivity discussion or narrative analysis comparing whether the overall compliance rates or documented biases significantly differed between the peer-reviewed papers and the unpublished dissertations.

8. Critical Misalignment Between Stated Objectives and Reported Results

Issue: A rigorous systematic review demands strict parallelism between the research objectives and the synthesized results. In this manuscript, there is a distinct structural mismatch.

1. While the objectives aim to assess regional compliance, the Results section merely lists individual study findings consecutively without aggregating or pooling the data into meaningful descriptive summaries (e.g., median compliance ranges).

2. The investigation of "determinants and barriers" promised in the framework is presented without a structured thematic coding system, making the narrative synthesis appear anecdotal rather than systematic.

3. Any comparative objective across different West African settings is entirely neglected in the results, despite the inclusion of both peer-reviewed papers and grey literature from two distinct countries (Ghana and Nigeria).

Recommendation: The authors must drastically restructure the Results section to mirror the specific objectives point-by-point. They should utilize explicit subheadings in the Results that correspond directly to each research question, and ensure that data from all 10 included studies are synthesized collectively under those specific themes rather than reported as isolated study summaries.

9. Superficial and Unstructured Discussion Section

Issue: The Discussion section is currently underdeveloped and mirrors a superficial repetition of the findings rather than offering a critical, contextualized synthesis. Because the Results lack a clear comparative and thematic structure, the Discussion fails to deeply interpret **why** OHS compliance rates and barriers differ or align between the two distinct nations (Ghana and Nigeria). It lacks a broader epidemiological and public health contextualization regarding the socio-economic, political, or legislative infrastructures governing the construction sectors in these specific West African settings.

Recommendation: The Discussion must be thoroughly revised. The authors should:

1. Move beyond merely restating the numbers and focus on interpreting the underlying systemic, institutional, or behavioral reasons behind the documented OHS compliance rates.

2. Provide a robust, critical comparison explicitly discussing how the differences in national occupational health policies, enforcement mechanisms, or socioeconomic contexts between Ghana and Nigeria could explain the variances in the study outcomes.

### Minor Concerns

1. Table Condensation and Formatting

Issue: Splitting the descriptive results into 5 separate, consecutive tables (Tables 3, 4, 5, 6, and 7) creates unnecessary fragmentation and heavily disrupts scannability. Furthermore, some cells and column boundaries contain truncated text or awkward spacing.

Recommendation: I strongly recommend consolidating Tables 3, 4, 5, and 6 into a single, cohesive "Domain-Specific OHS Compliance Findings" matrix table. This will allow readers to easily evaluate and cross-compare different compliance domains across the 10 included studies.

2. Abstract Refinement

Issue: The abstract mentions that the search ran through *"February 2025"*, but lines 130 and 141 specify the absolute cutoff date as *"12th February 2025"*.

Recommendation: Ensure absolute alignment of dates.

3. Unexplained Terminology

Issue: On line 239, the authors use the phrase *"DIK1-classified contractors in Ghana"*. This localized classification system will not be understood by a global public health audience.

Recommendation: Provide a brief parenthetical clarification explaining what a "DIK1" classification signifies (e.g., large-scale/financial capacity tier) within the Ghanaian construction ecosystem.
