# Supplementary material for: Occupational health and safety standards compliance among construction workers in West Africa: A systematic review of available evidence from Ghana and Nigeria
Source: PLOS Glob Public Health. 2026 Nov 13;6(11):e0006724. doi: 10.1371/journal.pgph.0006724 (PMC13766276; doi:10.1371/journal.pgph.0006724)
Supplement: Supplementary file 2 [file pgph.0006724.s008.pdf]

Response to Editor and Reviewers

Manuscript ID: PGPH-D-26-02159

Revised title: Occupational Health and Safety Standards Compliance among Construction Workers in West Africa: A Systematic Review of Available Quantitative Evidence from Ghana and Nigeria

Journal: PLOS Global Public Health

Dear Dr. Maung Maung and Reviewers,

We thank the Academic Editor and both reviewers for their detailed and constructive comments. We have substantially revised the manuscript. The principal changes include clarification of the West Africa-wide review scope and Ghana/Nigeria evidence base, an updated literature search through 18 August 2026, addition of two eligible low/moderate-risk Nigerian studies to the primary synthesis, a complete source-level data audit, removal of review-generated composite compliance scores, operational separation of PPE use, access and provision, organisational/regulatory implementation, knowledge/training and welfare measures, expanded JBI decision rules and sensitivity analyses, restructuring of the Results around the research questions and a substantially revised Discussion that distinguishes evidence, interpretation and recommendation. Our point-by-point responses are provided below.

Responses to the Academic Editor / Journal Requirements

1. Update the literature search

Response: We agree and updated the search through 18 August 2026 using the same information sources and eligibility criteria as the original review. The update identified 219

records. After removal of 65 duplicates, 154 records were screened; 130 were excluded at title/abstract screening and 24 underwent full-text assessment. Twenty reports were excluded at full text, leaving four newly eligible studies. Two were judged at high risk of bias and excluded from the primary synthesis, while Ameen et al. (2026) and Moda et al. (2026) were added. The primary synthesis therefore increased from 10 to 12 studies (seven Ghana; five Nigeria). Across the original and updated searches, 2,724 records were identified, 759 duplicates were removed, 1,965 records were screened, 247 reports underwent full-text assessment, 17 studies met eligibility criteria, five high-risk studies were excluded from the primary synthesis and 12 studies remained. The Abstract, Methods, Results, PRISMA flow, tables, Discussion and Conclusion were updated. The two new studies expanded the Nigerian evidence but did not materially reverse the principal interpretation. The evidence remained heterogeneous and geographically concentrated in Ghana and Nigeria.

2. Data availability requirements for systematic reviews

Response: We acknowledge the journal's requirement for reproducible underlying review data. The Supporting Information package is specified as Supplementary Appendix 4 and includes: (i) the complete record-level identification/screening log across the original and updated searches, including duplicate and exclusion status; (ii) itemized full-text exclusion reasons; (iii) the source-verified extraction dataset with extractor names and extraction/verification dates; (iv) documentation of missing, unclear or internally inconsistent data and how they were handled. Access information for non-peer-reviewed sources were also provided. The Data Availability statement in the manuscript has been changed from 'available upon reasonable request' to a Supporting Information-based statement. The complete Supplementary Appendix 4 will be uploaded with the revised submission so that these underlying review data are freely available in accordance with PLOS policy.

All 17 eligible studies, including the five high-risk studies excluded from the primary synthesis can be found in Supplementary Appendix 2-3.

3. Financial Disclosure

Response: The Financial Disclosure has been revised exactly as requested: 'The authors received no specific funding for this work.' The same wording is included in the manuscript.

4. Competing Interests

Response: The Competing Interests statement has been revised exactly as requested: 'The authors have declared that no competing interests exist.' The same wording is included in the manuscript.

5. Data Availability statement

Response: The previous statement that data were available from the corresponding author upon reasonable request has been removed. The revised manuscript states: 'All data underlying the findings reported in this systematic review are provided within the manuscript and its Supporting Information files, including the study-screening data, full-text exclusion reasons, extracted study data and risk-of-bias assessments.'

6. Separate figure files

Response: The PRISMA flow diagram has been updated to reflect the combined original and updated search counts. The figure is supplied separately as a TIFF file, and the embedded figure has been removed from the revised manuscript while the figure caption is retained.

7. Reviewer-suggested citations

Response: Neither reviewer required citation of a specific previously published work. We therefore did not add references solely to satisfy a citation request. Additional literature was

added only where independently relevant to strengthen the knowledge-gap and regulatory-context discussion.

Responses to Reviewer 1

Major Comment 1 - Title inconsistency and scope definition

Response: We agree that the submitted title was internally inconsistent. The title has been revised to: 'Occupational Health and Safety Standards Compliance among Construction Workers in West Africa: A Systematic Review of Available Quantitative Evidence from Ghana and Nigeria.' We deliberately retain 'West Africa' to reflect the prespecified review/search scope while explicitly stating that the eligible evidence identified was from Ghana and Nigeria. Throughout the manuscript, statements have been revised to distinguish the regional scope from the geographic distribution of the evidence and to avoid generalizing Ghana/Nigeria findings to all West Africa.

Major Comment 2 - Knowledge gap and rationale in the Introduction

Response: The Introduction has been substantially rewritten. It now distinguishes this review from prior reviews of construction safety research in developing countries, occupational injuries and African construction regulation; explains the rationale for a West Africa-wide evidence map; identifies the measurement, geographic and evidence-to-policy gaps addressed by the review; and closes with explicit public-health and policy relevance. We have avoided claiming that no related review exists and instead state the narrower gap: no identified review had systematically synthesized quantitative empirical compliance-related measures across West Africa in the manner undertaken here.

Major Comment 3 - Study selection and conflict resolution

Response: The Methods now state that two reviewers independently screened titles/abstracts and full texts. Any disagreement at either screening stage was directly adjudicated by a third independent reviewer. The same direct third-reviewer adjudication procedure is described for risk-of-bias disagreements. Data extraction was also conducted independently by two reviewers, with discrepancies discussed and resolved by rechecking the primary source.

Major Comment 4 - Search strategy and potential missing studies

Response: The Methods and Supplementary Appendix 1 now clarify that the geographic search component included individual names of all 16 West African countries in addition to regional descriptors. Google Scholar was searched through Harzing's Publish or Perish, and all returned records were exported for screening. The search was updated through 18 August 2026. The English-language restriction is now explicitly acknowledged in the Discussion as a potential source of geographic selection bias, particularly for Francophone and Lusophone West Africa. The omission of Scopus, Web of Science and Embase is also acknowledged as a limitation.

Major Comment 5 - Data synthesis and statistical analysis

Response: The synthesis section has been rewritten in operational detail. Meta-analysis was considered inappropriate because outcome definitions, PPE items, denominators, units of analysis and statistical reporting were substantially heterogeneous. A structured narrative synthesis was conducted across four predefined OHS compliance-related domains: PPE use/access/provision; regulatory and organisational implementation; safety knowledge/awareness/training; and welfare-facility provision/use. Study-specific estimates were retained, comparable constructs were summarized descriptively where defensible, non-

equivalent constructs were not combined, missing data were not imputed and no review-generated composite compliance score was calculated.

Major Comment 6 - Inconsistencies between text and tables

Response: We fully agree and conducted a source-level audit of the primary studies. The revised manuscript corrects sample sizes, denominators, labels and construct classifications. Examples include distinguishing Boakye et al.'s 5.8% earplug access from actual use, treating conditional PPE-use percentages only among workers with access, correcting the mislabelled 65.6% 'safety belt' value, separating Rockson's distinct company-practice response categories, correcting Friday and Chika's training-source percentages, and removing all review-generated composite scores. The consolidated Table 3 now preserves the original construct and denominator rather than presenting unlike measures as a common compliance metric.

Major Comment 7 - Grey literature versus peer-reviewed evidence

Response: We agree with the need to assess robustness by publication status. After the search update, the primary synthesis includes seven peer-reviewed studies and five non-peer-reviewed sources. We also corrected the description of the non-peer-reviewed group: it comprises four postgraduate theses/dissertations and one conference proceeding, rather than five theses/dissertations. A publication-status sensitivity analysis compares all 12 studies with the seven peer-reviewed studies alone. Restricting to peer-reviewed evidence did not materially change the qualitative direction of findings across PPE, organisational implementation, knowledge/training and welfare domains. Publication status was not used as a proxy for methodological quality; JBI appraisal was considered separately.

Major Comment 8 - Alignment between objectives and Results

Response: The aim and objectives have been revised and the Results have been restructured to mirror them directly. The review now asks: (1) what quantitative evidence is available on

OHS compliance-related outcomes; (2) what factors are associated with those outcomes; and (3) what strategies were reported or proposed for improvement. The Results are organized under these research questions rather than consecutive study summaries. Because no intervention study was included, the manuscript no longer claims to evaluate effective strategies. Country patterns are discussed descriptively and cautiously rather than treated as a causal Ghana-versus-Nigeria comparison.

Major Comment 9 - Discussion

Response: The Discussion has been substantially rewritten. It now interprets the measurement heterogeneity, distinguishes behavioural compliance from access/provision/knowledge, incorporates the two updated studies, contextualizes Ghana and Nigeria using current national/ILO information, discusses public-health implications of PPE and welfare gaps, and separates evidence from contextual inference and policy/research recommendations. National regulatory differences are presented as context only and are not used to causally explain between-study differences.

Minor Comment 1 - Table condensation and formatting

Response: Tables 3-6 from the submitted manuscript have been consolidated into a single 'Domain-specific OHS compliance-related findings' matrix (revised Table 3). The former factors/strategies table is retained as revised Table 4. The new matrix explicitly distinguishes use, access, provision, knowledge/training and welfare measures and is formatted for cross-study comparison.

Minor Comment 2 - Abstract search date

Response: The Abstract, Methods and supplementary search documentation now use the exact final updated-search date of 18 August 2026. The original search end date (12 February 2025) is retained only when describing the original search phase.

Minor Comment 3 - D1K1 terminology

Response: The localized contractor classification has been clarified. Where relevant, D denotes building works and K denotes civil works, while the numerical class denotes contractor capacity, with Class 1 representing the highest classification. The revised manuscript avoids using the term without explanation; the supporting Ghana Ministry source has been added to the reference list.

Responses to Reviewer 2

Comment 1 - Title and geographic scope

Response: We agree that the submitted title was confusing. We revised it to 'Occupational Health and Safety Standards Compliance among Construction Workers in West Africa: A Systematic Review of Available Quantitative Evidence from Ghana and Nigeria.' We retained West Africa because it reflects the prespecified review/search scope, but the title and manuscript now explicitly identify Ghana and Nigeria as the countries contributing eligible primary-synthesis evidence. We no longer state or imply that findings from these two countries represent all West Africa.

Comment 2 - Cross-sectional evidence and strategies

Response: We fully agree. All studies retained in the primary synthesis are cross-sectional and none evaluates an intervention. The objectives and research questions have therefore been revised to synthesize quantitative compliance-related evidence, identify factors associated with compliance-related outcomes, and summarize strategies reported or proposed by primary-study authors. The manuscript no longer claims to assess effective strategies. The protocol's additional interest in the impact of non-compliance is addressed transparently: the updated search identified one cross-sectional study reporting associations between training/PPE and injury, but no causal impact is inferred.

Comment 3 - Google Scholar screening and database coverage

Response: Google Scholar was searched through Harzing's Publish or Perish in three batches because of query-length limitations. In the original search, the batches returned 980, 829 and 509 records (2,318 total); the updated batches returned 70, 50 and 17 records (137 total). Every record returned was exported into the screening dataset; the search was not restricted to the first 100, 200 or another arbitrary ranked subset. We did not add Scopus, Web of Science or Embase. Their omission is now explicitly acknowledged as a limitation. The search was nevertheless updated through 18 August 2026 using all original information sources.

Comment 4 - JBI decision rule and high-risk exclusions

Response: We agree that the submitted Methods did not adequately describe the decision rule. The revised Methods now state how each of the eight JBI items was judged (Yes/No/Unclear/Not applicable) and clarify that no numerical score or percentage threshold was used. Overall Low/Moderate/High judgments were review-specific domain-based judgments that considered the pattern and methodological importance of concerns. No single failed item automatically resulted in exclusion. Seventeen studies met eligibility criteria; five were judged high risk and excluded from the primary synthesis. Key concerns among high-risk studies involved participant selection, validity/reliability of exposure or outcome measurement, standardisation, confounding and analytical validity. We also disclose that automatic exclusion of high-risk studies was not explicitly prespecified in the protocol and therefore constitutes a protocol deviation. A sensitivity analysis that reintroduced all five high-risk studies did not materially reverse the principal domain-level interpretation.

Comment 6 - Operational definition of compliance

Response: We fully agree. The revised manuscript no longer treats PPE use, access, employer provision, knowledge, training, inspections and welfare facilities as equivalent. Worker

behavioural compliance is distinguished from organisational/regulatory implementation. PPE use, access and source/provision are separate constructs. Safety knowledge, awareness and training are synthesized as compliance-related or enabling measures rather than direct behavioural compliance. Welfare availability/provision is distinguished from adequacy/use where possible. These distinctions are reflected in the Methods, Results and revised Table 3, and all review-generated composite compliance scores have been removed.

Comment 7 - Boakye safety-belt inconsistency

Response: The reviewer is correct. Rechecking the primary study showed that 42.1% referred to 'always' use of a safety harness/belt among the 19 workers who had access to one, whereas the previously reported 65.6% was the sum of 'always' (59.0%) and 'often' (6.6%) use for safety boots/shoes among workers with access. The 65.6% value had been incorrectly labelled as safety-belt use in our submitted manuscript. This has been corrected throughout. We now retain the original denominators and explicitly label use frequencies as conditional on access.

Comment 8 - Causal wording

Response: We agree. The Abstract, Results, Discussion and Conclusion were reviewed for causal language. Phrases such as 'leads to', 'results in', 'root cause', 'determines' and causal use of 'influences' have been removed or replaced with non-causal language such as 'was associated with', 'was reported as a barrier', 'may reflect' or 'was proposed'. The limitations now explicitly emphasize that cross-sectional designs cannot establish temporality or causality and that most outcomes were self-reported.

Comment 9 - Balance in the Discussion

Response: The Discussion now explicitly separates three levels: evidence directly reported by included studies; contextual interpretation or inference, signaled with cautious language; and

policy/practice/research recommendations. Proposed strategies are not described as demonstrated effective interventions. The Conclusion is similarly restricted to claims supported by the available cross-sectional evidence.

We again thank the Editor and Reviewers for their comments. We believe the revisions have substantially improved the methodological transparency, internal consistency and interpretive precision of the manuscript.

Sincerely,

Kimblyn Anim, on behalf of all authors
