# Supplementary material for: Occupational health and safety standards compliance among construction workers in West Africa: A systematic review of available evidence from Ghana and Nigeria
Source: PLOS Glob Public Health. 2026 Nov 13;6(11):e0006724. doi: 10.1371/journal.pgph.0006724 (PMC13766276; doi:10.1371/journal.pgph.0006724)
Supplement: Supplementary file 3 [file pgph.0006724.s009.pdf]

Response to the Academic Editor and Reviewers

PLOS Global Public Health | PGPH-D-26-02159R1

Dear Dr. Maung Maung and Reviewers,

Thank you for the further evaluation and constructive feedback. We are encouraged that Reviewer #1 considers the earlier comments addressed and that Reviewer #3 finds the review substantially improved. We have undertaken a further consistency and language audit of the clean manuscript and generated a new tracked-changes copy relative to the last submitted clean manuscript. The revision does not change the identified studies, eligibility rules, numerical results or narrative sensitivity analyses; it clarifies their interpretation and corrects overstatements.

Academic Editor

Comment: The authors have addressed previous comments constructively and comprehensively, but remaining internal inconsistencies and wording issues require correction.

Response: We agree. We reconciled the study counts and study-selection terminology in the Abstract, Methods and Results; aligned the Abstract conclusion with the domain-specific Conclusions; qualified geographic evidence-gap claims; and corrected statements that inappropriately combined self-reported and observed information or overstated causal or intervention evidence. The manuscript states that 17 studies were eligible, five were assessed at high risk of bias and excluded from the primary synthesis, and 12 were retained (seven Ghanaian and five Nigerian). The PRISMA flow counts remain 2,724 records, 759 duplicates, 1,965 screened, 247 full texts assessed, 230 excluded and 17 eligible.

Reviewer #1

Comment: "All comments have been addressed."

Response: We thank Reviewer #1 for reviewing the previous revision and for the positive assessment.

Reviewer #3

1. Comment: The previous marked-up manuscript appeared to retain "120 methodologically sound studies," although the primary synthesis had 12 studies, and displayed an older data-availability statement alongside the revised one.

Response: Thank you for identifying this. We confirm that the primary synthesis contains 12 studies; 17 met eligibility criteria and five were rated at high risk of bias and excluded from the primary synthesis (but reintroduced in sensitivity analysis). The preceding clean manuscript stated 12, while the previously uploaded marked-up file displayed superseded deleted numerals alongside inserted revisions. We have prepared a fresh, separately tracked version based directly on the last submitted clean manuscript, so the current tracked file no longer carries the prior revision history.

The clean manuscript contains one active data-availability statement: "All data underlying the findings reported in this systematic review are provided within the manuscript and its supporting information files, including the study-screening data, full-text exclusion reasons, extracted study data and risk of bias assessments." The previous "available upon reasonable request" wording is not part of the clean or new tracked manuscript. We will ensure the same statement is entered in the submission-system Data Availability field.

2. Comment: Do not describe all included studies as uniformly "methodologically sound," given that the primary synthesis includes low- and moderate-risk studies.

Response: Agreed. The Strengths section now describes the evidence as "12 cross-sectional studies assessed as having low or moderate risk of bias".

3. Comment: An apparent absence of methodologically sound evidence in all other countries cannot be inferred from searches restricted to English and omitting Scopus, Web of Science and Embase.

Response: We agree. The revised Abstract and Results now specify that no eligible low- or moderate-risk studies from the other 14 countries were identified by the searches and eligibility criteria used in this review. The Geographic and evidence gaps section explicitly states that this finding does not demonstrate the absence of relevant or rigorous research from those countries. The Limitations section acknowledge that the language restriction and omitted databases may have missed eligible studies, particularly in Francophone and Lusophone settings. We have avoided extrapolating the Ghanaian and Nigerian findings to all West African construction workers.

4. Comment: The overall conclusion should emphasise heterogeneous gaps across compliance-related domains instead of assigning one overall compliance category.

Response: Agreed. The revised Abstract conclusion now describes heterogeneous gaps in selected PPE practices, organisational implementation and welfare facilities rather than a regional "low to moderate" compliance level. The Discussion and Conclusion distinguish worker practices, PPE access and provision, respondent perceptions, safety knowledge and training, and welfare facilities. We retain separate study-specific results without generating an overall pooled compliance estimate or equating nonidentical constructs.

5. Comment: Remaining language should accurately reflect what is supported by the available cross-sectional evidence.

Response: We made several additional precision edits. The Limitations now recognise the presence of direct worker- and site-observation data alongside self-reports.

We appreciate the opportunity to revise the manuscript and hope that these targeted corrections address the remaining concerns.

Sincerely,

Kimblyn Anim

On behalf of all co-authors
