# Supplementary material for: Occupational health and safety standards compliance among construction workers in West Africa: A systematic review of available evidence from Ghana and Nigeria
Source: PLOS Glob Public Health. 2026 Nov 13;6(11):e0006724. doi: 10.1371/journal.pgph.0006724 (PMC13766276; doi:10.1371/journal.pgph.0006724)
Supplement: Supplementary file 4 [file pgph.0006724.s001.docx]

**COMPLIANCE WITH OCCUPATIONAL HEALTH AND SAFETY STANDARDS AMONG CONSTRUCTION WORKERS IN WEST AFRICA: A SYSTEMATIC REVIEW PROTOCOL**

Kimblyn Anim^1^, Saphiel Osei Poku^2^, Glypsy Agyiri-Kyereme^3,4^

^1^Department of Urban Roads, Ashanti Region, Ministry of Roads and Highways, Ghana

^2^Department of Optometry and Visual Science, Faculty of Biosciences, College of Science, Kwame Nkrumah University of Science and Technology, Kumasi, Ghana

^3^ Faculty of Health and Medical Sciences, Asante Akyem Campus, Presbyterian University, Ghana

^4^ Nyinahin Community Hospital, Nyinahin, Ashanti Region, Ghana

Correspondence:

Kimblyn Anim

Department of Urban Roads, Ashanti Region

Ministry of Roads and Highways

Ghana

Email: [kimblyna14@gmail.com](mailto:kimblyna14@gmail.com)

Tel: +233248649487 / +233506771585

**Abstract**

**Background:** The construction industry remains one of the most hazardous occupational sectors globally, with workers exposed to physical, chemical, ergonomic, biological, psychosocial, and accident-related hazards. In West Africa, occupational health and safety challenges may be intensified by labour-intensive construction methods, developing economies, informal employment arrangements, limited enforcement capacity, and fragmented regulatory systems. Although individual studies have examined occupational health and safety practices among construction workers, evidence on compliance with occupational health and safety standards in West Africa has not been systematically synthesized.

**Objective:** This systematic review protocol describes the planned methods for identifying, appraising, and synthesizing evidence on compliance with occupational health and safety standards among construction workers in West Africa. The review will also examine factors influencing compliance, the impact of non-compliance on workers’ health and safety, and strategies proposed or used to improve compliance.

**Methods:** The review will follow PRISMA-P guidance for systematic review protocols and the final review will be reported according to PRISMA 2020. The PECOS framework will guide eligibility criteria. Eligible studies will include quantitative studies involving construction workers in West African countries and reporting occupational health and safety compliance-related outcomes. PubMed, IEEE Xplore, EBSCOhost, ScienceDirect, and Google Scholar will be searched for studies published in English from June 2001 to February 2025. Two reviewers will independently screen titles, abstracts, and full texts, extract data, and assess methodological quality. The Joanna Briggs Institute critical appraisal checklist will be used for cross-sectional studies, and ROBINS-I will be used for eligible non-randomized studies where applicable. Disagreements will be resolved through discussion or consultation with a third reviewer. A narrative synthesis will be conducted, with findings organized by compliance domains, influencing factors, health and safety consequences, and strategies for improving compliance.

**Discussion:** This review is expected to provide region-specific evidence on occupational health and safety compliance among construction workers in West Africa. The findings may inform policymakers, employers, regulators, worker organizations, and researchers by identifying compliance gaps, vulnerable worker groups, and priority areas for intervention and future research.

**Keywords:** occupational health and safety; construction workers; compliance; West Africa; systematic review protocol; PPE; workplace safety.

**Background**

Occupational health and safety is also a science focused on ensuring safety in the workplace (Tamene et al., 2020). Many research studies have highlighted the benefits of occupational health and safety practices such as effective application of ergonomic principles in workplaces, machine design, job design, environment and facilities design (Ashraf & Biman, 1995; Biman, 1987; Biman & Arijit, 1996; Catchpole et al., 2021; Das & Shikdar, 1999; George & Martin, 1991; Resnick & Zanotti, 1997).

55,000 fatal injuries occur each year in the construction industry (ElSafty et al., 2012) which is due to their exposure to health risks or hazards related to their work (Ambrosini, 2007; ElSafty et al., 2012; Ngaruiya et al., 2019; Sherratt et al., 2013). The prevalent hazards in the construction industry are in the form of radiation and noise termed as physical hazards as well as asbestos and disinfectants called chemical hazards. Other hazards include ergonomic hazards manifesting in the form of poor work postures, irregular work situations such as night work, raising of bulky equipment, irregular workdays and workplace violence such as harassment (Al-Rawajfeh & Tarawneh, 2020). The direct and indirect costs of both fatal and non-fatal construction injuries have been estimated to be over 10 billion USD per year globally (Gittleman et al., 2010).

As the world evolves, there is a continuous change in occupational health and safety issues which poses a new challenge for its practice in most of the African countries because the tool and expertise needed to deal with such problems are not yet advanced in most African countries as compared to the developed countries (Takele Tadesse, 2006). Occupational health and safety issues are predominant in most African countries due to inadequate attention given to occupational health and safety by the industries and governments (Myburgh et al., 2004). Other reasons include developing economies, poor cultures in relation to health and safety issues and competing political, social and economic interests (Cao et al., 2021; Gbadago et al., 2017).

**Rationale for this systematic review**

As an effort to prioritize occupational health and safety, management systems for occupational health and safety have been adopted by the construction industry (Nikolaou, 2016; Yoon et al., 2013). Despite the extensive measures to ensure workplace safety, occupational health and safety issues still remains high in the construction industry with accidents continuing to occur often with significant human and economic consequences (Väyrynen et al., 2015). The construction industry still remains one of the most dangerous industries despite the fact that many companies in the sector have successfully adopted occupational health and safety management systems to address potential accidents and injuries (Ghodrati et al., 2018). Sub-Saharan Africa has the highest rate per worker of occupational injuries (Lund & Marriott, 2011). West African countries are all classified as either low income or lower middle income countries (World Bank, 2024) and their developing economies partially accounts for the predominance of occupational health and safety issues.

It has been well documented that, personal and work characteristics such as non-compliance to safety regulations combined with the working environment and organizational factors influence the creation of a hazardous environment that can result in accidents and ill-health (Cheng et al., 2012; Chi et al., 2013; Dumrak et al., 2013). Rigorous adherence to health and safety regulations can significantly reduce the risk of accidents and protect workers from harm (Emmeline de Chazal, 2024). As such, this systematic review aims to analyze and synthesize the existing research on the compliance level of construction workers with occupational health and safety standards in West Africa aiming to improve safety outcomes. By putting evidence on the compliance with occupational health and safety standards among construction workers in West Africa, the review seeks to provide region-specific insights on occupational health and safety standards in West Africa. It also seeks to improve the long-term health and quality of life of construction workers. The review will aim to recommend strategies for stricter enforcement of occupational health and safety regulations and practices in West Africa.

**Review Questions**

- What are the key factors that influence compliance with occupational health and safety standards among construction workers in West Africa?
- What is the impact of non-compliance with occupational health and safety standards on the health and safety of construction workers in West Africa?
- What are the effective strategies for improving compliance with occupational health and safety standards in West Africa?

**Methods**

The review will adhere to the guidelines outlined in the Preferred Reporting Items for Systematic Review and Meta-Analysis extension for protocols (PRISMA-P) checklist (Shamseer et al., 2015) (***S1 Table***) and similarly the full review report will follow the PRISMA checklist (Page et al., 2021). To ensure a structured approach we will follow the guidance specified in the Cochrane Handbook (Higgins et al., 2023) and utilize the PECOS framework to clearly define our Population, Exposure, Comparator, Outcomes and Study designs relevant to our review. The databases and the flow of studies will be reported using PRISMA Flow Diagram (Page et al., 2021) (***S1 Fig)***.

**Criteria for including studies for this review**

***Population***

Studies involving participants of all age groups and diverse ethnic populations who work in a construction setting in West Africa will be eligible for inclusion in this review.

***Exposure***

We would explore studies that focus on occupational health and safety in the construction industry including workplace hazards such as physical, chemical, ergonomic, musculoskeletal, psychological and biological hazards as well as occupational health and safety regulations, measures and compliance.

***Comparator***

Not applicable

***Outcomes***

***Primary outcome***

1. Analyze and synthesize the existing research on the compliance level of construction workers with occupational health and safety standards in West Africa aiming to improve safety outcomes.

***Secondary outcome***

1. To identify factors that influence compliance with occupational health and safety standards among construction workers in West Africa.
2. To analyze effective strategies for improving compliance with occupational health and safety standards

***Study designs***

We will include Quantitative studies, including randomized and non-randomized study designs (i.e. cross-sectional, case-control and cohort observational studies). Qualitative study designs, mixed methods study designs, abstracts, case studies, case series, commentaries, reviews, protocols and editorial letters will be excluded.

**Search methods for identification of studies**

***Electronic databases***

Comprehensive searches will be conducted in PubMed, IEEE Xplore, EBSCOhost, Google Scholar and Science direct for relevant studies published from June 2001 to 12^th^ February 2025 with language limited to English. We will use the following search terms ‘Compliance’, nested with all applicable alternate terms and ‘Occupational health and safety’, nested with all applicable alternate terms and ‘Construction workers’, ‘building workers’, nested with all applicable alternate terms and ‘West Africa’, ‘Ecowas region’, nested with all applicable alternate terms. See Table for details on search strategy.

A detailed search strategy developed for all databases.

**Screening and selection of studies**

All retrieved articles from the searches will be organised and deduplicated using EndNote. The deduplicated studies will then be exported to Rayyan (a web-based systematic review application) (Ouzzani et al., 2016) where screening and selection of studies will be conducted. Two of the reviewers will independently screen the titles and abstracts for eligible literature according to the selection criteria. The eligible studies will further be selected with full text screening after removing duplications. The inclusion criteria are as follows: (1) No restriction regarding age, race and gender (2) Articles dated from 6^th^ June 2001 to 12^th^ February 2025 (3) Quantitative studies such as randomized and non-randomized study designs (i.e Cross-sectional, case-control and cohort observational studies) (4) Studies written in English. Our exclusion criteria are qualitative and mixed methods study designs, studies which are focused on regions outside west Africa and studies that only provide abstracts, editorials or commentary articles. Any disagreements will be resolved through a third reviewer. The study selection process and the various decisions for inclusion and exclusion of studies will be summarized and represented using PRISMA flow diagram (***S1 Fig***) (Shamseer et al., 2015). All studies excluded after the full text evaluation will be listed in a table and the reasons for exclusion provided.

**Data extraction and management**

Relevant data from the included studies will be extracted independently by two reviewers on to Microsoft Excel spreadsheet. Randomly selected studies will be pretested to ensure consistency during the extraction process. The data to be extracted will include study identification (study ID, author, year of publication, study title), study characteristics (study design, study location, study population, study demographics including age, gender, occupation), factors that influence compliance with occupational health and safety standards among construction workers in West Africa, effective strategies for improving compliance with occupational health and safety standards among construction workers in West Africa, study limitations, assessment of study quality.

**Assessment of quality of included studies**

The Joanna Briggs Institute (JBI) checklist for analytical cross-sectional studies will be used to assess the risk of bias of the included cross-sectional studies (P-F, 2020). We will categorize the judgments as low, moderate or high risk of bias. Risk of bias in non-randomized studies of Interventions (ROBINS-I) tool (Sterne et al., 2021) will be used to assess the risk of bias in observation quantitative studies. Seven domains of bias that would be assessed are confounding bias, participant selection bias, interventions classification bias, deviations from intended interventions bias, missing data bias, measurement of outcomes bias and selection of the reported result bias. We will rate the risk of bias as low, moderate, serious, or critical. The quality of the studies included will be carried out by two reviewers independently. Any conflict will be resolved through a third reviewer.

**Data analysis**

A narrative synthesis approach will be adopted to analyze and summarize the findings from the included studies. The extracted data will be synthesized and organised based on relevant study characteristics. A descriptive analysis will be performed to summarize the study characteristics, participant demographics, factors that influence compliance with occupational health and safety standards among construction workers in West Africa, impact of non-compliance with occupational health and safety standards on the health and safety of construction workers in West Africa, effective strategies for improving compliance with occupational health and safety standards among construction workers in West Africa. The findings will be synthesized and interpreted in the context of existing knowledge, with representative quotes illustrating key themes.

**Dealing with missing data**

The pattern of missing data and the potential impact on the study findings and trustworthiness of the conclusions will be explored as well as reasons for the missing data. Efforts will be made to contact the corresponding authors of the studies to request the missing information or data.

**Ethical considerations and dissemination**

This is a secondary research based on published studies or publicly available data, therefore, no ethical approval is required. However, eligible studies with serious ethical issues will be excluded and the reasons for exclusion documented. The final review report will be in the form of a scientific paper for submission for publication in high impact factor peer-reviewed journal.

**Discussion**

This systematic review is motivated by the pressing necessity to fill the clear void in the literature concerning the compliance with occupational health and safety standards among construction workers in West Africa. Despite the increasing recognition of construction workplace safety including adherence to occupational health and safety regulations, a comprehensive evaluation of the compliance level in West Africa is deficient. This protocol seeks to address this gap by providing a detailed plan for synthesizing existing evidence and generating findings regarding the compliance with occupational health and safety standards among construction workers in West Africa.

In this systematic review, a detailed plan of all the steps of the review including; eligibility criteria, information sources, search strategy, study selection, data extraction, quality assessment and data synthesis has been described. This careful approach will ensure the production of unbiased evidence regarding the compliance with occupational health and safety standards among construction workers in West Africa. By following the structured methodology, we aim to provide reliable findings into reducing morbidity and mortality in the construction sector, produce better health outcomes and present better health statistics in the construction industry. The knowledge obtained from this review will serve as a valuable resource for the construction industry, policymakers and researchers working to make the construction workplace as safe as possible.

**Strengths**

This systematic review will endeavor to compile and refine empirical findings to the utmost degree employing explicit, transparent and replicable approaches to address the review questions, guaranteeing meticulous adherence to best practices in evidence synthesis. The procedure entails a thorough search strategy executed across multiple databases, to attempt to retrieve all relevant studies.

**Limitations**

Our review will systematically search articles published in selected major databases. According to our preset inclusion and exclusion criteria, we will not specifically review conferences and abstracts that may contain the new policies and trends in the compliance with occupational health and safety standards among construction workers in West Africa. Our review focusing on only quantitative studies may lead to incomplete understanding of factors affecting compliance and may skew the interpretation of the search results. Only English articles will be included in this review, and thus relevant studies published in other languages may be missed.

**Supporting information**

***S1 Fig.*** PRISMA-P 2020 Flow Diagram

***S1 Table.*** PRISMA-P 2015 checklist

**References**

Al-Rawajfeh, M. A., & Tarawneh, S. (2020). Impact of applying occupational safety and health programs on employees performance in construction sector. *Solid State Technology*, *63*(6), 3332-3346.

Ambrosini, M. (2007). Employment and working conditions of migrant workers-Italy.

Ashraf, A. S., & Biman, D. (1995). A field study of worker productivity improvements. *Applied Ergonomics*, *26*(1), 21-27. <https://doi.org/https://doi.org/10.1016/0003-6870(95)95748-O>

Biman, D. (1987). An ergonomic approach to designing a manufacturing work system. *International Journal of Industrial Ergonomics*, *1*(3), 231-240. <https://doi.org/https://doi.org/10.1016/0169-8141(87)90017-5>

Biman, D., & Arijit, K. S. (1996). Industrial workstation design: A systematic ergonomics approach. *Applied Ergonomics*, *27*(3), 157-163. <https://doi.org/https://doi.org/10.1016/0003-6870(96)00008-7>

Cao, Z., Chen, T., & Cao, Y. (2021). Effect of Occupational Health and Safety Training for Chinese Construction Workers Based on the CHAID Decision Tree. *Front Public Health*, *9*, 623441. <https://doi.org/10.3389/fpubh.2021.623441>

Catchpole, K., Bowie, P., Fouquet, S., Rivera, J., & Hignett, S. (2021). Frontiers in human factors: embedding specialists in multi-disciplinary efforts to improve healthcare. *Int J Qual Health Care*, *33*(Supplement_1), 13-18. <https://doi.org/10.1093/intqhc/mzaa108>

Cheng, C. W., Leu, S. S., Cheng, Y. M., Wu, T. C., & Lin, C. C. (2012). Applying data mining techniques to explore factors contributing to occupational injuries in Taiwan's construction industry. *Accid Anal Prev*, *48*, 214-222. <https://doi.org/10.1016/j.aap.2011.04.014>

Chi, S., Han, S., & Kim, D. Y. (2013). Relationship between unsafe working conditions and workers’ behavior and impact of working conditions on injury severity in US construction industry. *Journal of construction engineering and management*, *139*(7), 826-838.

Das, B., & Shikdar, A. A. (1999). Participative versus assigned production standard setting in a repetitive industrial task: a strategy for improving worker productivity. *Int J Occup Saf Ergon*, *5*(3), 417-430. <https://doi.org/10.1080/10803548.1999.11076429>

Dumrak, J., Mostafa, S., Kamardeen, I., & Rameezdeen, R. (2013). Factors associated with the severity of construction accidents: The case of South Australia. *Australasian Journal of Construction Economics and Building, The*, *13*(4), 32-49.

ElSafty, A., ElSafty, A., & Malek, M. (2012). Construction safety and occupational health education in Egypt, the EU, and US firms. *Open journal of civil engineering*, *2*(3), 174-182.

Emmeline de Chazal. (2024). *Health & Safety in Construction: All You Need to Know*. <https://www.skillcast.com/blog/health-safety-construction-everything-you-need-know>

Gbadago, P., Amedome, S. N., & Honyenuga, B. Q. (2017). The impact of occupational health and safety measures on employee performance at the South Tongu District Hospital. *Global Journal Inc.(USA)*, *17*(5), 13-19.

George, J. B., & Martin, G. H. (1991). A field study of productivity improvements in the manufacturing of circuit boards. *International Journal of Industrial Ergonomics*, *7*(3), 207-215. <https://doi.org/https://doi.org/10.1016/0169-8141(91)90004-6>

Ghodrati, N., Yiu, T. W., Wilkinson, S., & Shahbazpour, M. (2018). A new approach to predict safety outcomes in the construction industry. *Safety science*, *109*, 86-94.

Gittleman, J. L., Gardner, P. C., Haile, E., Sampson, J. M., Cigularov, K. P., Ermann, E. D., Stafford, P., & Chen, P. Y. (2010). [Case Study] CityCenter and Cosmopolitan Construction Projects, Las Vegas, Nevada: lessons learned from the use of multiple sources and mixed methods in a safety needs assessment. *J Safety Res*, *41*(3), 263-281. <https://doi.org/10.1016/j.jsr.2010.04.004>

Higgins, J., Thomas, J., Chandler, J., Cumpston, M., Li, T., Page, M., & Welch, V. (2023). Cochrane handbook for systematic reviews of interventions version 6.4 (updated August 2023). Cochrane, 2023. *Available from training. cochrane. org/handbook*.

Lund, F., & Marriott, A. (2011). *Occupational health and safety and the poorest*. University of KwaZulu-Natal, School of Development Studies.

Myburgh, N. G., Hobdell, M. H., & Lalloo, R. (2004). African countries propose a regional oral health strategy: The Dakar Report from 1998. *Oral Dis*, *10*(3), 129-137. <https://doi.org/10.1046/j.1601-0825.2003.01008.x>

Ngaruiya, F. W., Ogendi, G. M., & Mokua, M. A. (2019). Occupational health risks and hazards among the fisherfolk in Kampi Samaki, Lake Baringo, Kenya. *Environmental Health Insights*, *13*, 1178630219881463.

Nikolaou, I. E. (2016). Occupational health and safety within corporate social responsibility context: a balanced scorecard dynamic decision making model. *International Journal of Decision Support Systems*, *2*(1-3), 54-70.

Ouzzani, M., Hammady, H., Fedorowicz, Z., & Elmagarmid, A. (2016). Rayyan—a web and mobile app for systematic reviews. *Systematic Reviews*, *5*(1), 210. <https://doi.org/10.1186/s13643-016-0384-4>

P-F, M. S. M. Z. T. C. A. E. S. K. S. R. C. M. Q. R. M. P. L. K. M. (2020). Chapter 7: Systematic reviews of etiology and risk. In A. E. M. Z (Ed.), *JBI Manual for Evidence Synthesis*. JBI.

Page, M. J., Moher, D., Bossuyt, P. M., Boutron, I., Hoffmann, T. C., Mulrow, C. D., Shamseer, L., Tetzlaff, J. M., Akl, E. A., Brennan, S. E., Chou, R., Glanville, J., Grimshaw, J. M., Hróbjartsson, A., Lalu, M. M., Li, T., Loder, E. W., Mayo-Wilson, E., McDonald, S., . . . McKenzie, J. E. (2021). PRISMA 2020 explanation and elaboration: updated guidance and exemplars for reporting systematic reviews. *BMJ*, *372*, n160. <https://doi.org/10.1136/bmj.n160>

Resnick, M. L., & Zanotti, A. (1997). Using ergonomics to target productivity improvements. *Computers & Industrial Engineering*, *33*(1), 185-188. <https://doi.org/https://doi.org/10.1016/S0360-8352(97)00070-3>

Shamseer, L., Moher, D., Clarke, M., Ghersi, D., Liberati, A., Petticrew, M., Shekelle, P., & Stewart, L. A. (2015). Preferred reporting items for systematic review and meta-analysis protocols (PRISMA-P) 2015: elaboration and explanation. *Bmj*, *349*.

Sherratt, F., Farrell, P., & Noble, R. (2013). UK construction site safety: discourses of enforcement and engagement. *Construction management and economics*, *31*(6), 623-635.

Sterne, J., Hernán, M., & Reeves, B. (2021). Risk of bias in nonrandomized studies of interventions (ROBINS-I). *Accessed December*, *15*.

Takele Tadesse, M. A. (2006). *Occupational Health and Safety* <https://www.cartercenter.org/resources/pdfs/health/ephti/library/lecture-notes/env-occupational-health-students/ln-occ-health-safety-final.pdf>

Tamene, A., Afework, A., & Mebratu, L. (2020). A Qualitative Study of Barriers to Personal Protective Equipment Use among Laundry Workers in Government Hospitals, Hawassa, Ethiopia. *J Environ Public Health*, *2020*, 5146786. <https://doi.org/10.1155/2020/5146786>

Väyrynen, S., Häkkinen, K., & Niskanen, T. (2015). *Integrated Occupational Safety and Health Management*. Springer.

World Bank. (2024). *World Bank Country and Lending Groups*. <https://datahelpdesk.worldbank.org/knowledgebase/articles/906519-world-bank-country-and-lending-groups>

Yoon, S. J., Lin, H. K., Chen, G., Yi, S., Choi, J., & Rui, Z. (2013). Effect of occupational health and safety management system on work-related accident rate and differences of occupational health and safety management system awareness between managers in South Korea's construction industry. *Safety and health at work*, *4*(4), 201-209.

***S1Table***. PRISMA-P (Preferred Reporting Items for Systematic Review and Meta-Analysis Protocols) 2015 checklist: recommended items to address in a systematic review protocol*

| **Section and topic** | **Item No** | **Checklist item** |
| --- | --- | --- |
| **ADMINISTRATIVE INFORMATION** | | |
| Title: |  |  |
| Identification | 1a | Identify the report as a protocol of a systematic review |
| Update | 1b | If the protocol is for an update of a previous systematic review, identify as such |
| Registration | 2 | If registered, provide the name of the registry (such as PROSPERO) and registration number |
| Authors: |  |  |
| Contact | 3a | Provide name, institutional affiliation, e-mail address of all protocol authors; provide physical mailing address of corresponding author |
| Contributions | 3b | Describe contributions of protocol authors and identify the guarantor of the review |
| Amendments | 4 | If the protocol represents an amendment of a previously completed or published protocol, identify as such and list changes; otherwise, state plan for documenting important protocol amendments |
| Support: |  |  |
| Sources | 5a | Indicate sources of financial or other support for the review |
| Sponsor | 5b | Provide name for the review funder and/or sponsor |
| Role of sponsor or funder | 5c | Describe roles of funder(s), sponsor(s), and/or institution(s), if any, in developing the protocol |
| **INTRODUCTION** | | |
| Rationale | 6 | Describe the rationale for the review in the context of what is already known |
| Objectives | 7 | Provide an explicit statement of the question(s) the review will address with reference to participants, interventions, comparators, and outcomes (PICO) |
| **METHODS** | | |
| Eligibility criteria | 8 | Specify the study characteristics (such as PICO, study design, setting, time frame) and report characteristics (such as years considered, language, publication status) to be used as criteria for eligibility for the review |
| Information sources | 9 | Describe all intended information sources (such as electronic databases, contact with study authors, trial registers or other grey literature sources) with planned dates of coverage |
| Search strategy | 10 | Present draft of search strategy to be used for at least one electronic database, including planned limits, such that it could be repeated |
| Study records: |  |  |
| Data management | 11a | Describe the mechanism(s) that will be used to manage records and data throughout the review |
| Selection process | 11b | State the process that will be used for selecting studies (such as two independent reviewers) through each phase of the review (that is, screening, eligibility and inclusion in meta-analysis) |
| Data collection process | 11c | Describe planned method of extracting data from reports (such as piloting forms, done independently, in duplicate), any processes for obtaining and confirming data from investigators |
| Data items | 12 | List and define all variables for which data will be sought (such as PICO items, funding sources), any pre-planned data assumptions and simplifications |
| Outcomes and prioritization | 13 | List and define all outcomes for which data will be sought, including prioritization of main and additional outcomes, with rationale |
| Risk of bias in individual studies | 14 | Describe anticipated methods for assessing risk of bias of individual studies, including whether this will be done at the outcome or study level, or both; state how this information will be used in data synthesis |
| Data synthesis | 15a | Describe criteria under which study data will be quantitatively synthesised |
|  | 15b | If data are appropriate for quantitative synthesis, describe planned summary measures, methods of handling data and methods of combining data from studies, including any planned exploration of consistency (such as I^2^, Kendall’s τ) |
|  | 15c | Describe any proposed additional analyses (such as sensitivity or subgroup analyses, meta-regression) |
|  | 15d | If quantitative synthesis is not appropriate, describe the type of summary planned |
| Meta-bias(es) | 16 | Specify any planned assessment of meta-bias(es) (such as publication bias across studies, selective reporting within studies) |
| Confidence in cumulative evidence | 17 | Describe how the strength of the body of evidence will be assessed (such as GRADE) |

* It is strongly recommended that this checklist be read in conjunction with the PRISMA-P Explanation and Elaboration (cite when available) for important clarification on the items. Amendments to a review protocol should be tracked and dated. The copyright for PRISMA-P (including checklist) is held by the PRISMA-P Group and is distributed under a Creative Commons Attribution Licence 4.0.

*From: Shamseer L, Moher D, Clarke M, Ghersi D, Liberati A, Petticrew M, Shekelle P, Stewart L, PRISMA-P Group. Preferred reporting items for systematic review and meta-analysis protocols (PRISMA-P) 2015: elaboration and explanation. BMJ. 2015 Jan 2;349(jan02 1):g7647*

| **Section and topic** | **Item No** | **Checklist item** |
| --- | --- | --- |
| **ADMINISTRATIVE INFORMATION** | | |
| Title: |  |  |
| Identification | 1a | Identify the report as a protocol of a systematic review |
| Update | 1b | If the protocol is for an update of a previous systematic review, identify as such |
| Registration | 2 | If registered, provide the name of the registry (such as PROSPERO) and registration number |
| Authors: |  |  |
| Contact | 3a | Provide name, institutional affiliation, e-mail address of all protocol authors; provide physical mailing address of corresponding author |
| Contributions | 3b | Describe contributions of protocol authors and identify the guarantor of the review |
| Amendments | 4 | If the protocol represents an amendment of a previously completed or published protocol, identify as such and list changes; otherwise, state plan for documenting important protocol amendments |
| Support: |  |  |
| Sources | 5a | Indicate sources of financial or other support for the review |
| Sponsor | 5b | Provide name for the review funder and/or sponsor |
| Role of sponsor or funder | 5c | Describe roles of funder(s), sponsor(s), and/or institution(s), if any, in developing the protocol |
| **INTRODUCTION** | | |
| Rationale | 6 | Describe the rationale for the review in the context of what is already known |
| Objectives | 7 | Provide an explicit statement of the question(s) the review will address with reference to participants, interventions, comparators, and outcomes (PICO) |
| **METHODS** | | |
| Eligibility criteria | 8 | Specify the study characteristics (such as PICO, study design, setting, time frame) and report characteristics (such as years considered, language, publication status) to be used as criteria for eligibility for the review |
| Information sources | 9 | Describe all intended information sources (such as electronic databases, contact with study authors, trial registers or other grey literature sources) with planned dates of coverage |
| Search strategy | 10 | Present draft of search strategy to be used for at least one electronic database, including planned limits, such that it could be repeated |
| Study records: |  |  |
| Data management | 11a | Describe the mechanism(s) that will be used to manage records and data throughout the review |
| Selection process | 11b | State the process that will be used for selecting studies (such as two independent reviewers) through each phase of the review (that is, screening, eligibility and inclusion in meta-analysis) |
| Data collection process | 11c | Describe planned method of extracting data from reports (such as piloting forms, done independently, in duplicate), any processes for obtaining and confirming data from investigators |
| Data items | 12 | List and define all variables for which data will be sought (such as PICO items, funding sources), any pre-planned data assumptions and simplifications |
| Outcomes and prioritization | 13 | List and define all outcomes for which data will be sought, including prioritization of main and additional outcomes, with rationale |
| Risk of bias in individual studies | 14 | Describe anticipated methods for assessing risk of bias of individual studies, including whether this will be done at the outcome or study level, or both; state how this information will be used in data synthesis |
| Data synthesis | 15a | Describe criteria under which study data will be quantitatively synthesised |
|  | 15b | If data are appropriate for quantitative synthesis, describe planned summary measures, methods of handling data and methods of combining data from studies, including any planned exploration of consistency (such as I^2^, Kendall’s τ) |
|  | 15c | Describe any proposed additional analyses (such as sensitivity or subgroup analyses, meta-regression) |
|  | 15d | If quantitative synthesis is not appropriate, describe the type of summary planned |
| Meta-bias(es) | 16 | Specify any planned assessment of meta-bias(es) (such as publication bias across studies, selective reporting within studies) |
| Confidence in cumulative evidence | 17 | Describe how the strength of the body of evidence will be assessed (such as GRADE) |

* It is strongly recommended that this checklist be read in conjunction with the PRISMA-P Explanation and Elaboration (cite when available) for important clarification on the items. Amendments to a review protocol should be tracked and dated. The copyright for PRISMA-P (including checklist) is held by the PRISMA-P Group and is distributed under a Creative Commons Attribution Licence 4.0.

*From: Shamseer L, Moher D, Clarke M, Ghersi D, Liberati A, Petticrew M, Shekelle P, Stewart L, PRISMA-P Group. Preferred reporting items for systematic review and meta-analysis protocols (PRISMA-P) 2015: elaboration and explanation. BMJ. 2015 Jan 2;349(jan02 1):g7647*

***S1 Fig***. PRISMA 2020 flow diagram for new systematic reviews which included searches of databases and registers only.

**Identification of studies via databases and registers**

Records removed *before screening*:

Duplicate records removed (n = )

Records marked as ineligible by automation tools (n = )

Records removed for other reasons (n = )

Records identified from*:

Databases (n = )

Registers (n = )

**Identification**

Records screened

(n = )

Records excluded**

(n = )

Reports sought for retrieval

(n = )

Reports not retrieved

(n = )

**Screening**

Reports assessed for eligibility

(n = )

Reports excluded:

Reason 1 (n = )

Reason 2 (n = )

Reason 3 (n = )

etc.

Studies included in review

(n = )

Reports of included studies

(n = )

**Included**
