# Supplementary material for: Occupational health and safety standards compliance among construction workers in West Africa: A systematic review of available evidence from Ghana and Nigeria
Source: PLOS Glob Public Health. 2026 Nov 13;6(11):e0006724. doi: 10.1371/journal.pgph.0006724 (PMC13766276; doi:10.1371/journal.pgph.0006724)
Supplement: Supplementary file 5 [file pgph.0006724.s002.docx]

**Search strategies employed in this systematic review**

**Search in EBSCOhost**:

| 1 | (Compliance OR Adherence OR Abidance OR Conformance) | n = 1,149,816 |
| --- | --- | --- |
| 2 | (Occupational Health and Safety OR ohs OR workplace safety OR safety regulations OR safety protocols OR safety measures OR occupational safety standards OR occupational safety regulations OR safety culture OR safety and health guidelines) | n = 372,865 |
| 3 | (construction workers OR construction laborers OR construction personnel OR construction workforce OR building workers OR site workers OR construction employees) | n = 77,240 |
| 4 | (west africa OR ecowas region OR niger OR burkina faso OR mali OR ghana OR togo OR nigeria OR benin OR liberia OR côte d’ivoire OR the gambia OR sierra leone OR senegal OR guinea OR guinea bissau OR cabo verde OR cape verde OR ivory coast OR mauritania OR cameroon) | n = 1,039,274 |
| 5 | Search: (#1) AND (#2) AND (#3) AND (#4) | **n = 6** |
|  | Updated search from 02/2025 to 18^th^ August 2026 | **n = 12** |
|  | Total | **N = 18** |

**Search in IEEE Xplore:**

| (("All Metadata":compliance) OR ("All Metadata":adherence) OR ("All Metadata":abidance) OR ("All Metadata":conformance)) AND (("All Metadata":occupational health and safety) OR ("All Metadata":ohs) OR ("All Metadata":workplace safety) OR ("All Metadata":safety regulations) OR ("All Metadata":safety protocols) OR ("All Metadata":safety measures) OR ("All Metadata":occupational safety standards) OR ("All Metadata":occupational health and safety regulations) OR ("All Metadata":safety culture) OR ("All Metadata":occupational health and safety guidelines)) AND (("All Metadata":construction workers) OR ("All Metadata":construction laborers) OR ("All Metadata":construction personnel) OR ("All Metadata":construction workforce) OR ("All Metadata":building workers) OR ("All Metadata":site workers) OR ("All Metadata":construction employees)) AND (("All Metadata":west africa) OR ("All Metadata":ecowas region) OR ("All Metadata":niger) OR ("All Metadata":burkina faso) OR ("All Metadata":mali) OR ("All Metadata":ghana) OR ("All Metadata":togo) OR ("All Metadata":nigeria) OR ("All Metadata":benin) OR ("All Metadata":liberia) OR ("All Metadata":côte d’ivoire) OR ("All Metadata": ivory coast) OR ("All Metadata":the gambia) OR ("All Metadata":sierra leone) OR ("All Metadata":senegal) OR ("All Metadata":guinea) OR ("All Metadata":guinea bissau) OR ("All Metadata":cabo verde) OR ("All Metadata":cape verde) OR ("All Metadata":mauritania) OR ("All Metadata":cameroon)) | **n = 2** |
| --- | --- |
| Updated search from 02/2025 to 2026 | **n = 1** |
| Total | **N = 3** |

**Search in PubMed:**

| 1 | "compliances"[All Fields] OR "patient compliance"[MeSH Terms] OR ("patient"[All Fields] AND "compliance"[All Fields]) OR "patient compliance"[All Fields] OR "compliance"[All Fields] OR "compliance"[MeSH Terms] OR ("adherance"[All Fields] OR "adhere"[All Fields] OR "adhered"[All Fields] OR "adherence"[All Fields] OR "adherences"[All Fields] OR "adherent"[All Fields] OR "adherents"[All Fields] OR "adherer"[All Fields] OR "adherers"[All Fields] OR "adheres"[All Fields] OR "adhering"[All Fields]) OR "Abidance"[All Fields] OR ("conform"[All Fields] OR "conformability"[All Fields] OR "conformable"[All Fields] OR "conformality"[All Fields] OR "conformally"[All Fields] OR "conformance"[All Fields] OR "conformances"[All Fields] OR "conformant"[All Fields] OR "conformed"[All Fields] OR "conforming"[All Fields] OR "conformism"[All Fields] OR "conformities"[All Fields] OR "conformity"[All Fields] OR "conforms"[All Fields] OR "molecular conformation"[MeSH Terms] OR ("molecular"[All Fields] AND "conformation"[All Fields]) OR "molecular conformation"[All Fields] OR "conformal"[All Fields]) | n = 1301307 |
| --- | --- | --- |
| 2 | "occup health saf"[Journal] OR ("occupational"[All Fields] AND "health"[All Fields] AND "and"[All Fields] AND "safety"[All Fields]) OR "occupational health and safety"[All Fields] OR "ohs"[All Fields] OR (("workplace"[MeSH Terms] OR "workplace"[All Fields] OR "workplaces"[All Fields] OR "workplace s"[All Fields]) AND ("safety"[MeSH Terms] OR "safety"[All Fields] OR "safeties"[All Fields])) OR (("safety"[MeSH Terms] OR "safety"[All Fields] OR "safeties"[All Fields]) AND ("legislation and jurisprudence"[MeSH Subheading] OR ("legislation"[All Fields] AND "jurisprudence"[All Fields]) OR "legislation and jurisprudence"[All Fields] OR "regulations"[All Fields] OR "social control, formal"[MeSH Terms] OR ("social"[All Fields] AND "control"[All Fields] AND "formal"[All Fields]) OR "formal social control"[All Fields] OR "regulate"[All Fields] OR "regulates"[All Fields] OR "regulating"[All Fields] OR "regulation s"[All Fields] OR "regulative"[All Fields] OR "regulator"[All Fields] OR "regulator s"[All Fields] OR "regulators"[All Fields] OR "regulated"[All Fields] OR "regulation"[All Fields])) OR (("safety"[MeSH Terms] OR "safety"[All Fields] OR "safeties"[All Fields]) AND ("protocol"[All Fields] OR "protocol s"[All Fields] OR "protocolized"[All Fields] OR "protocols"[All Fields])) OR (("safety"[MeSH Terms] OR "safety"[All Fields] OR "safeties"[All Fields]) AND ("measurability"[All Fields] OR "measurable"[All Fields] OR "measurably"[All Fields] OR "measure s"[All Fields] OR "measureable"[All Fields] OR "measured"[All Fields] OR "measurement"[All Fields] OR "measurement s"[All Fields] OR "measurements"[All Fields] OR "measurer"[All Fields] OR "measurers"[All Fields] OR "measuring"[All Fields] OR "measurings"[All Fields] OR "measurment"[All Fields] OR "measurments"[All Fields] OR "weights and measures"[MeSH Terms] OR ("weights"[All Fields] AND "measures"[All Fields]) OR "weights and measures"[All Fields] OR "measure"[All Fields] OR "measures"[All Fields])) OR (("occupational health"[MeSH Terms] OR ("occupational"[All Fields] AND "health"[All Fields]) OR "occupational health"[All Fields] OR ("occupational"[All Fields] AND "safety"[All Fields]) OR "occupational safety"[All Fields]) AND ("reference standards"[MeSH Terms] OR ("reference"[All Fields] AND "standards"[All Fields]) OR "reference standards"[All Fields] OR "standardization"[All Fields] OR "standard"[All Fields] OR "standard s"[All Fields] OR "standardisation"[All Fields] OR "standardisations"[All Fields] OR "standardise"[All Fields] OR "standardised"[All Fields] OR "standardises"[All Fields] OR "standardising"[All Fields] OR "standardization s"[All Fields] OR "standardizations"[All Fields] OR "standardize"[All Fields] OR "standardized"[All Fields] OR "standardizes"[All Fields] OR "standardizing"[All Fields] OR "standards"[MeSH Subheading] OR "standards"[All Fields])) OR (("occupational health"[MeSH Terms] OR ("occupational"[All Fields] AND "health"[All Fields]) OR "occupational health"[All Fields] OR ("occupational"[All Fields] AND "safety"[All Fields]) OR "occupational safety"[All Fields]) AND ("legislation and jurisprudence"[MeSH Subheading] OR ("legislation"[All Fields] AND "jurisprudence"[All Fields]) OR "legislation and jurisprudence"[All Fields] OR "regulations"[All Fields] OR "social control, formal"[MeSH Terms] OR ("social"[All Fields] AND "control"[All Fields] AND "formal"[All Fields]) OR "formal social control"[All Fields] OR "regulate"[All Fields] OR "regulates"[All Fields] OR "regulating"[All Fields] OR "regulation s"[All Fields] OR "regulative"[All Fields] OR "regulator"[All Fields] OR "regulator s"[All Fields] OR "regulators"[All Fields] OR "regulated"[All Fields] OR "regulation"[All Fields])) OR ("safety management"[MeSH Terms] OR ("safety"[All Fields] AND "management"[All Fields]) OR "safety management"[All Fields] OR ("safety"[All Fields] AND "culture"[All Fields]) OR "safety culture"[All Fields]) OR (("safety"[MeSH Terms] OR "safety"[All Fields] OR "safeties"[All Fields]) AND ("health"[MeSH Terms] OR "health"[All Fields] OR "health s"[All Fields] OR "healthful"[All Fields] OR "healthfulness"[All Fields] OR "healths"[All Fields]) AND ("guideline"[Publication Type] OR "guidelines as topic"[MeSH Terms] OR "guidelines"[All Fields])) | n = 484051 |
| 3 | (("construct s"[All Fields] OR "constructed"[All Fields] OR "constructing"[All Fields] OR "construction"[All Fields] OR "constructions"[All Fields] OR "constructive"[All Fields] OR "constructively"[All Fields] OR "constructs"[All Fields] OR "dna, recombinant"[MeSH Terms] OR ("dna"[All Fields] AND "recombinant"[All Fields]) OR "recombinant dna"[All Fields] OR "construct"[All Fields]) AND ("occupational groups"[MeSH Terms] OR ("occupational"[All Fields] AND "groups"[All Fields]) OR "occupational groups"[All Fields] OR "worker"[All Fields] OR "workers"[All Fields] OR "worker s"[All Fields])) OR (("construct s"[All Fields] OR "constructed"[All Fields] OR "constructing"[All Fields] OR "construction"[All Fields] OR "constructions"[All Fields] OR "constructive"[All Fields] OR "constructively"[All Fields] OR "constructs"[All Fields] OR "dna, recombinant"[MeSH Terms] OR ("dna"[All Fields] AND "recombinant"[All Fields]) OR "recombinant dna"[All Fields] OR "construct"[All Fields]) AND ("labor s"[All Fields] OR "labored"[All Fields] OR "laborer"[All Fields] OR "laborer s"[All Fields] OR "laborers"[All Fields] OR "laboring"[All Fields] OR "labors"[All Fields] OR "labour"[All Fields] OR "work"[MeSH Terms] OR "work"[All Fields] OR "labor"[All Fields] OR "labor, obstetric"[MeSH Terms] OR ("labor"[All Fields] AND "obstetric"[All Fields]) OR "obstetric labor"[All Fields] OR "laboured"[All Fields] OR "labourer"[All Fields] OR "labourers"[All Fields] OR "labouring"[All Fields] OR "labours"[All Fields])) OR (("construct s"[All Fields] OR "constructed"[All Fields] OR "constructing"[All Fields] OR "construction"[All Fields] OR "constructions"[All Fields] OR "constructive"[All Fields] OR "constructively"[All Fields] OR "constructs"[All Fields] OR "dna, recombinant"[MeSH Terms] OR ("dna"[All Fields] AND "recombinant"[All Fields]) OR "recombinant dna"[All Fields] OR "construct"[All Fields]) AND ("occupational groups"[MeSH Terms] OR ("occupational"[All Fields] AND "groups"[All Fields]) OR "occupational groups"[All Fields] OR "personnel"[All Fields] OR "personnel s"[All Fields] OR "personnels"[All Fields])) OR (("construct s"[All Fields] OR "constructed"[All Fields] OR "constructing"[All Fields] OR "construction"[All Fields] OR "constructions"[All Fields] OR "constructive"[All Fields] OR "constructively"[All Fields] OR "constructs"[All Fields] OR "dna, recombinant"[MeSH Terms] OR ("dna"[All Fields] AND "recombinant"[All Fields]) OR "recombinant dna"[All Fields] OR "construct"[All Fields]) AND ("workforce"[MeSH Terms] OR "workforce"[All Fields] OR "workforces"[All Fields] OR "workforce s"[All Fields])) OR (("build"[All Fields] OR "building"[All Fields] OR "building s"[All Fields] OR "buildings"[All Fields] OR "builds"[All Fields]) AND ("occupational groups"[MeSH Terms] OR ("occupational"[All Fields] AND "groups"[All Fields]) OR "occupational groups"[All Fields] OR "worker"[All Fields] OR "workers"[All Fields] OR "worker s"[All Fields])) OR ("site"[All Fields] AND ("occupational groups"[MeSH Terms] OR ("occupational"[All Fields] AND "groups"[All Fields]) OR "occupational groups"[All Fields] OR "worker"[All Fields] OR "workers"[All Fields] OR "worker s"[All Fields])) OR (("construct s"[All Fields] OR "constructed"[All Fields] OR "constructing"[All Fields] OR "construction"[All Fields] OR "constructions"[All Fields] OR "constructive"[All Fields] OR "constructively"[All Fields] OR "constructs"[All Fields] OR "dna, recombinant"[MeSH Terms] OR ("dna"[All Fields] AND "recombinant"[All Fields]) OR "recombinant dna"[All Fields] OR "construct"[All Fields]) AND ("employee s"[All Fields] OR "occupational groups"[MeSH Terms] OR ("occupational"[All Fields] AND "groups"[All Fields]) OR "occupational groups"[All Fields] OR "employee"[All Fields] OR "employees"[All Fields])) | n = 178791 |
| 4 | "africa, western"[MeSH Terms] OR ("africa"[All Fields] AND "western"[All Fields]) OR "western africa"[All Fields] OR ("west"[All Fields] AND "africa"[All Fields]) OR "west africa"[All Fields] OR ("ecowas"[All Fields] AND ("geographic locations"[MeSH Terms] OR ("geographic"[All Fields] AND "locations"[All Fields]) OR "geographic locations"[All Fields] OR "region"[All Fields] OR "region s"[All Fields] OR "regional"[All Fields] OR "regionalization"[All Fields] OR "regionalizations"[All Fields] OR "regionalize"[All Fields] OR "regionalized"[All Fields] OR "regionalizing"[All Fields] OR "regionally"[All Fields] OR "regionals"[All Fields] OR "regions"[All Fields])) OR ("niger"[MeSH Terms] OR "niger"[All Fields]) OR ("burkina faso"[MeSH Terms] OR ("burkina"[All Fields] AND "faso"[All Fields]) OR "burkina faso"[All Fields]) OR ("mali"[MeSH Terms] OR "mali"[All Fields]) OR ("ghana"[MeSH Terms] OR "ghana"[All Fields] OR "ghana s"[All Fields]) OR ("togo"[MeSH Terms] OR "togo"[All Fields]) OR ("nigeria"[MeSH Terms] OR "nigeria"[All Fields] OR "nigeria s"[All Fields]) OR ("benin"[MeSH Terms] OR "benin"[All Fields] OR "benin s"[All Fields]) OR ("liberia"[MeSH Terms] OR "liberia"[All Fields] OR "liberia s"[All Fields]) OR ("cote"[All Fields] AND ("d ivoire"[All Fields] OR "d ivoire s"[All Fields])) OR ("gambia"[MeSH Terms] OR "gambia"[All Fields] OR "the gambia"[All Fields]) OR ("sierra leone"[MeSH Terms] OR ("sierra"[All Fields] AND "leone"[All Fields]) OR "sierra leone"[All Fields]) OR ("senegal"[MeSH Terms] OR "senegal"[All Fields] OR "senegal s"[All Fields]) OR ("guinea"[MeSH Terms] OR "guinea"[All Fields] OR "guinea s"[All Fields] OR "guineas"[All Fields]) OR ("guinea bissau"[MeSH Terms] OR "guinea bissau"[All Fields] OR ("guinea"[All Fields] AND "bissau"[All Fields]) OR "guinea bissau"[All Fields]) OR ("cabo verde"[MeSH Terms] OR ("cabo"[All Fields] AND "verde"[All Fields]) OR "cabo verde"[All Fields]) OR ("cabo verde"[MeSH Terms] OR ("cabo"[All Fields] AND "verde"[All Fields]) OR "cabo verde"[All Fields] OR ("cape"[All Fields] AND "verde"[All Fields]) OR "cape verde"[All Fields]) OR (("ivories"[All Fields] OR "ivory"[All Fields]) AND ("coast"[All Fields] OR "coast s"[All Fields] OR "coasts"[All Fields])) OR ("mauritania"[MeSH Terms] OR "mauritania"[All Fields]) OR ("cameroon"[MeSH Terms] OR "cameroon"[All Fields] OR "cameroons"[All Fields] OR "cameroon s"[All Fields]) | n = 399806 |
| 5 | Search: (#1) AND (#2) AND (#3) AND (#4) | **n = 28** |
|  | Updated search from 02/2025 to August 18, 2026 | **n = 14** |
|  | Total | **N = 42** |

**Search in Science Direct:** search done multiple times with different search terms because of limited words per search in the search engine.

| 1 | "compliance" AND ("occupational health and safety" OR "safety regulations" OR "safety guidelines") AND ("construction workers" OR "building workers" OR "site workers") AND "west africa" | n = 11 |
| --- | --- | --- |
|  | Updated search from 02/2025 to 2026 | n = 1 |
| 2 | "compliance" AND ("occupational health and safety" OR "safety regulations" OR "safety guidelines") AND ("construction workers" OR "building workers" OR "site workers") AND "ecowas region" | n = 0 |
|  | Updated search from 02/2025 to 2026 | n = 0 |
| 3 | "compliance" AND ("occupational health and safety" OR "safety regulations" OR "safety guidelines") AND ("construction workers" OR "building workers" OR "site workers") AND "niger" | n = 8 |
|  | Updated search from 02/2025 to 2026 | n = 1 |
| 4 | "compliance" AND ("occupational health and safety" OR "safety regulations" OR "safety guidelines") AND ("construction workers" OR "building workers" OR "site workers") AND "burkina faso" | n = 4 |
|  | Updated search from 02/2025 to 2026 | n = 1 |
| 5 | "compliance" AND ("occupational health and safety" OR "safety regulations" OR "safety guidelines") AND ("construction workers" OR "building workers" OR "site workers") AND "mali" | n = 4 |
|  | Updated search from 02/2025 to 2026 | n = 1 |
| 6 | "compliance" AND ("occupational health and safety" OR "safety regulations" OR "safety guidelines") AND ("construction workers" OR "building workers" OR "site workers") AND "ghana" | n = 30 |
|  | Updated search from 02/2025 to 2026 | n = 15 |
| 7 | "compliance" AND ("occupational health and safety" OR "safety regulations" OR "safety guidelines") AND ("construction workers" OR "building workers" OR "site workers") AND "togo" | n = 4 |
|  | Updated search from 02/2025 to 2026 | n = 1 |
| 8 | "compliance" AND ("occupational health and safety" OR "safety regulations" OR "safety guidelines") AND ("construction workers" OR "building workers" OR "site workers") AND "nigeria" | n = 46 |
|  | Updated search from 02/2025 to 2026 | n = 27 |
| 9 | "compliance" AND ("occupational health and safety" OR "safety regulations" OR "safety guidelines") AND ("construction workers" OR "building workers" OR "site workers") AND "benin" | n = 5 |
|  | Updated search from 02/2025 to 2026 | n = 1 |
| 10 | "compliance" AND ("occupational health and safety" OR "safety regulations" OR "safety guidelines") AND ("construction workers" OR "building workers" OR "site workers") AND "liberia" | n = 5 |
|  | Updated search from 02/2025 to 2026 | n = 0 |
| 11 | "compliance" AND ("occupational health and safety" OR "safety regulations" OR "safety guidelines") AND ("construction workers" OR "building workers" OR "site workers") AND "côte d’ivoire" | n = 0 |
|  | Updated search from 02/2025 to 2026 | n = 0 |
| 12 | "compliance" AND ("occupational health and safety" OR "safety regulations" OR "safety guidelines") AND ("construction workers" OR "building workers" OR "site workers") AND "ivory coast" | n = 2 |
|  | Updated search from 02/2025 to 2026 | n = 1 |
| 13 | "compliance" AND ("occupational health and safety" OR "safety regulations" OR "safety guidelines") AND ("construction workers" OR "building workers" OR "site workers") AND "the gambia" | n = 1 |
|  | Updated search from 02/2025 to 2026 | n = 0 |
| 14 | "compliance" AND ("occupational health and safety" OR "safety regulations" OR "safety guidelines") AND ("construction workers" OR "building workers" OR "site workers") AND "sierra leone" | n = 4 |
|  | Updated search from 02/2025 to 2026 | n = 0 |
| 15 | "compliance" AND ("occupational health and safety" OR "safety regulations" OR "safety guidelines") AND ("construction workers" OR "building workers" OR "site workers") AND "senegal" | n = 6 |
|  | Updated search from 02/2025 to 2026 | n = 2 |
| 16 | "compliance" AND ("occupational health and safety" OR "safety regulations" OR "safety guidelines") AND ("construction workers" OR "building workers" OR "site workers") AND "guinea" | n = 13 |
|  | Updated search from 02/2025 to 2026 | n = 3 |
| 17 | "compliance" AND ("occupational health and safety" OR "safety regulations" OR "safety guidelines") AND ("construction workers" OR "building workers" OR "site workers") AND "guinea bissau" | n = 0 |
|  | Updated search from 02/2025 to 2026 | n = 0 |
| 18 | "compliance" AND ("occupational health and safety" OR "safety regulations" OR "safety guidelines") AND ("construction workers" OR "building workers" OR "site workers") AND "cabo verde" | n = 0 |
|  | Updated search from 02/2025 to 2026 | n = 0 |
| 19 | "compliance" AND ("occupational health and safety" OR "safety regulations" OR "safety guidelines") AND ("construction workers" OR "building workers" OR "site workers") AND "cape verde" | n = 0 |
|  | Updated search from 02/2025 to 2026 | n = 0 |
| 20 | "compliance" AND ("occupational health and safety" OR "safety regulations" OR "safety guidelines") AND ("construction workers" OR "building workers" OR "site workers") AND "mauritania" | n = 2 |
|  | Updated search from 02/2025 to 2026 | n = 0 |
| 21 | "compliance" AND ("occupational health and safety" OR "safety regulations" OR "safety guidelines") AND ("construction workers" OR "building workers" OR "site workers") AND "cameroon"  Filtered from 2025 to 2026. N = 1 | n = 6 |
|  | Updated search from 02/2025 to 2026 | n = 1 |
| 22 | TOTAL | **n = 206** |

**Search in Google scholar using Harzing’s Publish or Perish:** search done multiple times with different search terms because of limited words per search in the search engine.

| 1 | "compliance" OR "adherence" "occupational health and safety" OR "safety regulations” OR "safety guidelines" "construction workers" OR "construction laborers" OR "building workers" OR "site workers" "west africa" OR "ecowas region" OR "niger" OR "burkina faso" OR "mali" OR "ghana" OR "togo" | n = 980 |
| --- | --- | --- |
|  | Updated search from 02/2025 to 18^th^ August, 2026 | n = 70 |
| 2 | "compliance" OR "adherence" "occupational health and safety" OR "safety regulations” OR "safety guidelines" "construction workers" OR "construction laborers" OR "building workers" OR "site workers" "nigeria" OR "benin" OR "liberia" OR "côte d’ivoire" OR "the gambia" OR "sierra leone" | n = 829 |
|  | Updated search from 02/2025 to 18^th^ August, 2026 | n = 50 |
| 3 | "compliance" OR "adherence" "occupational health and safety" OR "safety regulations” OR "safety guidelines" "construction workers" OR "construction laborers" OR "building workers" OR "site workers" "senegal" OR "guinea" OR "guinea bissau" OR "cabo verde" OR "mauritania" OR "cameroon" | n = 509 |
|  | Updated search from 02/2025 to 18^th^ August, 2026 | n = 17 |
| 4 | TOTAL | **n = 2455** |
