# Supplementary material for: Occupational health and safety standards compliance among construction workers in West Africa: A systematic review of available evidence from Ghana and Nigeria
Source: PLOS Glob Public Health. 2026 Nov 13;6(11):e0006724. doi: 10.1371/journal.pgph.0006724 (PMC13766276; doi:10.1371/journal.pgph.0006724)
Supplement: Supplementary file 6 [file pgph.0006724.s003.docx]

**Summary of risk of bias of peer-reviewed studies (JBI cross-sectional checklist)**

| Risk of Bias | Oluwafemi et al., (2017)  (1) | Afatsawu & Kheni (2022) | Boakye et al. (2022) | Friday & chika (2022) | Tannor et al. (2023) | Amadi & Udofia (2023) | Mukarram et al., (2024)  (2) |
| --- | --- | --- | --- | --- | --- | --- | --- |
| Clearly  Defined  Inclusion  Criteria |  |  |  |  |  |  |  |
| Detailed  Description of  Study subjects  & Settings |  |  |  |  |  |  |  |
| Validity &  Reliability of  Measured  Exposure |  |  |  |  |  |  |  |
| Standard Objective  Measurement  Of Condition |  |  |  |  |  |  |  |
| Confounding  Factors  Identified |  |  |  |  |  |  |  |
| Confounding Strategies  Stated |  |  |  |  |  |  |  |
| Validity &  Reliability of  Measured  Outcomes |  |  |  |  |  |  |  |
| Appropriate  Statistical analysis |  |  |  |  |  |  |  |
| Overall  Appraisal | High  Exclude | Moderate  Include | Moderate  Include | Low  Include | Low  Include | Low  Include | High  Exclude |

| Yes | No | Unclear | Not applicable |
| --- | --- | --- | --- |

**Summary of risk of bias of revised 4 newly added peer-reviewed studies (JBI cross-sectional checklist)**

| Risk of Bias | Ogunsusi et al. (2025) (3) | Ameen et al. (2026) | Moda et al. (2026) | Abiodun et al. (2025)(4) |
| --- | --- | --- | --- | --- |
| Clearly  Defined  Inclusion  Criteria |  |  |  |  |
| Detailed  Description of  Study subjects  & Settings |  |  |  |  |
| Validity &  Reliability of  Measured  Exposure |  |  |  |  |
| Standard Objective  Measurement  Of Condition |  |  |  |  |
| Confounding  Factors  Identified |  |  |  |  |
| Confounding Strategies  Stated |  |  |  |  |
| Validity &  Reliability of  Measured  Outcomes |  |  |  |  |
| Appropriate  Statistical analysis |  |  |  |  |
| Overall  Appraisal | High  Exclude | Low  Include | Moderate  Include | High  Exclude |

| Yes | No | Unclear | Not applicable |
| --- | --- | --- | --- |

**Summary of risk of bias of non peer-reviewed studies (JBI cross-sectional checklist)**

| Risk of Bias | Bonney (2016) | Agbede et al., (2016) | Rockson (2018) | Afia (2019) | Danquah (2019)  (5) | Hammond (2019) |
| --- | --- | --- | --- | --- | --- | --- |
| Clearly  Defined  Inclusion  Criteria |  |  |  |  |  |  |
| Detailed  Description of  Study subjects  & Settings |  |  |  |  |  |  |
| Validity &  Reliability of  Measured  Exposure |  |  |  |  |  |  |
| Standard Objective  Measurement  Of Condition |  |  |  |  |  |  |
| Confounding  Factors  Identified |  |  |  |  |  |  |
| Confounding Strategies  Stated |  |  |  |  |  |  |
| Validity &  Reliability of  Measured  Outcomes |  |  |  |  |  |  |
| Appropriate  Statistical analysis |  |  |  |  |  |  |
| Overall  Appraisal | Low  Include | Moderate  Include | Moderate  Include | Low  Include | High  Exclude | Low  Include |

| Yes | No | Unclear | Not applicable |
| --- | --- | --- | --- |

1.	Oluwafemi F, Abiola A, Akingbade A, ... Knowledge of Occupational Hazards, Attitude and Practice of Occupational Safety Measures Among Construction Workers in Different Building Sites Located in Ibeju …. Online journal of …. 2017.

2.	MUKARRAM M, MUHAMMAD I, ... ASSESSING THE LEVEL OF COMPLIANCE WITH HEALTH AND SAFETY STANDARDS ON SOME SELECTED CONSTRUCTION SITES IN KANO METROPOLIS …. … Journal of Built …. 2024.

3.	Oyekunle F, OGUNSUSI S, Aiyewalehinmi E, ... Assessment of occupational health and safety risks in selected construction sites in oyo state, Nigeria. International Journal of …. 2025.

4.	ABIODUN Y, OLUWASEUN A, OMOLARA O, ... EVALUATION OF SAFETY MEASURES, COMPLIANCE, AND COMPENSATION IN LAGOS STATE GOVERNMENT BUILDING CONSTRUCTION …2025.

5.	Danquah CO. Safety Management of Construction Projects: Practices, Constraints and Improvement Measures. Kumasi, Ghana: Kwame Nkrumah University of Science and Technology; 2019.
