# Supplementary material for: Occupational health and safety standards compliance among construction workers in West Africa: A systematic review of available evidence from Ghana and Nigeria
Source: PLOS Glob Public Health. 2026 Nov 13;6(11):e0006724. doi: 10.1371/journal.pgph.0006724 (PMC13766276; doi:10.1371/journal.pgph.0006724)
Supplement: Peer review history

# Peer review: Decision Letter 0

**DOI:** 10.1371/journal.pgph.0006724.r001

**Authors:** Theingi Maung Maung

8 Jul 2026

PGPH-D-26-02159

Occupational health and safety standards compliance among construction workers in Ghana: A systematic review evidence from Ghana and Nigeria

PLOS Global Public Health

Dear Dr. Anim,

Thank you for submitting your manuscript to PLOS Global Public Health. After careful consideration, we feel that it has merit but does not fully meet PLOS Global Public Health’s publication criteria as it currently stands. Therefore, we invite you to submit a revised version of the manuscript that addresses the points raised during the review process.

The study highlights important structural and behavioral issues, such as the knowledge-practice gap and the impact of precarious employment conditions on worker safety. However, several major methodological inconsistencies, severe data mismatches between the narrative text and tables, and geographical scope presentation issues must be resolved.

Please submit your revised manuscript by Sep 06 2026 11:59PM. If you will need more time than this to complete your revisions, please reply to this message or contact the journal office at globalpubhealth@plos.org. When you're ready to submit your revision, log on to https://www.editorialmanager.com/pgph/ and select the 'Submissions Needing Revision' folder to locate your manuscript file.

Please include the following items when submitting your revised manuscript:

- A letter that responds to each point raised by the editor and reviewer(s). You should upload this letter as a separate file labeled 'Response to Reviewers'.
- A marked-up copy of your manuscript that highlights changes made to the original version. You should upload this as a separate file labeled 'Revised Manuscript with Track Changes'.
- An unmarked version of your revised paper without tracked changes. You should upload this as a separate file labeled 'Manuscript'.

Guidelines for resubmitting your figure files are available below the reviewer comments at the end of this letter.

As the corresponding author, your ORCID iD is verified in the submission system and will appear in the published article. PLOS supports the use of ORCID, and we encourage all coauthors to register for an ORCID iD and use it as well. Please encourage your coauthors to verify their ORCID iD within the submission system before final acceptance, as unverified ORCID iDs will not appear in the published article. *Only*  the individual author can complete the verification step; PLOS staff *cannot*  verify ORCID iDs on behalf of authors.

We look forward to receiving your revised manuscript.

Kind regards,

Theingi Maung Maung, Ph.D

Academic Editor

PLOS Global Public Health

Journal Requirements:

1. We note that the most recent literature search was completed over a year before the manuscript was submitted to the journal. Systematic and scoping reviews should provide an up-to-date overview of the research topic covered. Therefore, we request that you please update your searches as part of your revision to ensure that the most up-to-date literature is included in your review, or provide scientific justification for not doing so. Please ensure you mention this in your response to reviewers, and discuss whether this has any impact on the Results and Conclusions of the study. If you are submitting a protocol, it is only necessary to state whether the searches will be updated prior to the publication of the results.

2. As required by our policy on Data Availability, please ensure your manuscript or supplementary information includes the following:

A numbered table of all studies identified in the literature search, including those that were excluded from the analyses.

For every excluded study, the table should list the reason(s) for exclusion.

If any of the included studies are unpublished, include a link (URL) to the primary source or detailed information about how the content can be accessed.

A table of all data extracted from the primary research sources for the systematic review and/or meta-analysis. The table must include the following information for each study:

Name of data extractors and date of data extraction

Confirmation that the study was eligible to be included in the review.

All data extracted from each study for the reported systematic review and/or meta-analysis that would be needed to replicate your analyses.

If data or supporting information were obtained from another source (e.g. correspondence with the author of the original research article), please provide the source of data and dates on which the data/information were obtained by your research group.

If applicable for your analysis, a table showing the completed risk of bias and quality/certainty assessments for each study or outcome.  Please ensure this is provided for each domain or parameter assessed. For example, if you used the Cochrane risk-of-bias tool for randomized trials, provide answers to each of the signalling questions for each study. If you used GRADE to assess certainty of evidence, provide judgements about each of the quality of evidence factor. This should be provided for each outcome.

An explanation of how missing data were handled.

This information can be included in the main text, supplementary information, or relevant data repository. Please note that providing these underlying data is a requirement for publication in this journal, and if these data are not provided your manuscript might be rejected.

3. Please amend your online Financial Disclosure statement. If you did not receive any funding for this study, please simply state: "The authors received no specific funding for this work."

4. Please update your online Competing Interests statement. If you have no competing interests to declare, please state: "The authors have declared that no competing interests exist."

5. In the online submission form, you indicated that:

"The datasets used and/or analysed during the current study are available from the corresponding author upon reasonable request."

All PLOS journals now require all data underlying the findings described in their manuscript to be freely available to other researchers, either

a) In a public repository,

b) Within the manuscript itself, or

c) Uploaded as supplementary information.

This policy applies to all data except where public deposition would breach compliance with the protocol approved by your research ethics board. If your data cannot be made publicly available for ethical or legal reasons (e.g., public availability would compromise patient privacy), please explain your reasons by return email and your exemption request will be escalated to the editor for approval. Your exemption request will be handled independently and will not hold up the peer review process, but will need to be resolved should your manuscript be accepted for publication. One of the Editorial team will then be in touch if there are any issues.

For further assistance, you may go to: http://journals.plos.org/globalpublichealth/s/data-availability

6. Please provide separate main figure files in .tif or .eps format only and remove any figures embedded in your manuscript file. Please also ensure that all files are under our size limit of 10MB. Please leave the figure captions in the manuscript.

For more information about how to convert your figure files please see our guidelines: https://journals.plos.org/globalpublichealth/s/figures

7. If the reviewer comments include a recommendation to cite specific previously published works, please review and evaluate these publications to determine whether they are relevant and should be cited. There is no requirement to cite these works unless the editor has indicated otherwise.

Additional Editor Comments (if provided):

[Note: HTML markup is below. Please do not edit.]

Reviewers' comments:

Reviewer's Responses to Questions

**Comments to the Author**

1. Does this manuscript meet PLOS Global Public Health’s publication criteria?

Reviewer #1: Yes

Reviewer #2: Yes

**********

2. Has the statistical analysis been performed appropriately and rigorously?

Reviewer #1: Yes

Reviewer #2: N/A

**********

3. Have the authors made all data underlying the findings in their manuscript fully available (please refer to the Data Availability Statement at the start of the manuscript PDF file)?

The PLOS Data policy

Reviewer #1: Yes

Reviewer #2: Yes

**********

4. Is the manuscript presented in an intelligible fashion and written in standard English?

Reviewer #1: Yes

Reviewer #2: Yes

**********

Reviewer #1: Please find the detailed comments in the attached document, 'Reviewer Comments.pdf.

General Evaluation

This manuscript addresses a critical occupational health gap within the West African construction sector, specifically synthesizing compliance with occupational health and safety (OHS) standards in Ghana and Nigeria. The study highlights important structural and behavioral issues, such as the knowledge-practice gap and the impact of precarious employment conditions on worker safety. However, several major methodological inconsistencies, severe data mismatches between the narrative text and tables, and geographical scope presentation issues must be resolved before this manuscript can be considered for publication.

### Major Concerns

1. Title Inconsistency and Scope Definition

Issue: There is a logical contradiction and redundancy in the current title: "Occupational health and safety standards compliance among construction workers in Ghana: A systematic review evidence from Ghana and Nigeria." The primary title restricts the geographic focus strictly to Ghana, which directly contradicts the methodology and inclusion criteria that encompass the wider West African region (specifically extracting data from both Ghana and Nigeria).

Recommendation: The title should be streamlined to reflect the actual regional scope defined in the methodology without adding redundant country clauses. The authors are recommended to revise the title to something more concise and professional, such as:

1: "Occupational health and safety standards compliance among construction workers in West Africa: A systematic review"

2: "Compliance with occupational health and safety standards among West African construction workers: A systematic review"

2. Weak Justification of the Knowledge Gap and Rationale in the Introduction

Issue: The Introduction currently provides a broad overview of the construction industry and OHS general statistics, but it fails to articulate a compelling "Knowledge Gap." It remains unclear whether previous systematic reviews on OHS compliance in developing countries or Africa exist, and if so, what specific limitations they possess that this study aims to rectify. Furthermore, the selection of the "West African" regional scope is introduced without a clear underlying rationale or contextual justification (e.g., shared regulatory frameworks, economic ties, or common labor market characteristics).

Recommendation: The authors need to heavily strengthen the Introduction by addressing the following points:

1. Explicitly state what is uniquely missing from current global or regional OHS literature regarding construction workers.

2. Provide a clear rationale for choosing West Africa as the defined scope. Explain the socio-economic or regulatory commonalities of this region that justify examining them under a unified systematic review.

3. Conclude the Introduction with a clear statement regarding how this synthesis will translate into global public health actions or regional policy-making, aligning better with the scope of PLOS Global Public Health.

3. Incomplete Description of the Study Selection and Conflict Resolution Process in Methods

Issue: In the Methods section, the authors state that two independent reviewers conducted the screening and selection of articles. While utilizing two reviewers meets the minimum standard for a systematic review, the manuscript completely lacks a detailed explanation of the "Conflict Resolution Process." It is not specified how disagreements between the two primary reviewers regarding study eligibility or quality assessment were resolved.

4. Potential Missing Studies & Search Strategy Limits

Issue: The PECOS framework defines the targeted population as "all age groups... who work in a construction setting in West Africa". The authors note that no eligible studies were identified from the other 14 West African nations. However, it is unclear if individual country names (e.g., Liberia, Sierra Leone, Senegal) were explicitly included in the search strings, or if only regional umbrella terms ("West Africa", "ECOWAS") were used.

Recommendation: Please display the precise search string used for at least one major database (e.g., PubMed) in the main text or appendix to prove the sensitivity of the search. Additionally, since many West African nations do not use English as their official language (e.g., countries using French or Portuguese), the strict English-language restriction should be explicitly addressed in the Discussion as a limitation regarding potential geographic selection bias.

5. Vague and Unstructured Data Synthesis and Statistical Analysis Section

Issue: The "Data synthesis and statistical analysis" section is currently very superficial and lacks operational detail. While the authors state that a "narrative synthesis" was conducted due to the heterogeneity of the data, they fail to specify the structured framework used to guide this synthesis (e.g., whether the synthesis was structured by compliance domains, geographic locations, or study designs). Merely summarizing the findings of individual studies consecutively does not constitute a rigorous systematic narrative synthesis.

6. Severe Inconsistencies and Mismatches Between Text and Tables

Issue: There are multiple conflicting statistical values and reporting discrepancies between the text and the corresponding data tables. For example:

1. Page 16 (Lines 269–271): The text states, "Boakye et al. (2022) reported that 70% of Ghanaian workers used earplugs." However, looking at Table 4 (Page 22) under the Boakye et al. (2022) row, it reports: "Access to PPEs: ... Earplug (5.8%)". Access cannot logically be lower than actual usage.

2. Page 40 (Line 335): The narrative points out that welfare facilities were among only 9.3% of practices in Rockson (2018). Yet, in Table 4 (Page 21), the entry for Rockson (2018) claims: "Provision of PPE 27.9%".

Recommendation: The authors must perform a rigorous data audit across the entire manuscript to ensure that every percentage, sample size, and finding cited in the narrative exactly mirrors the data extracted into Tables 2 through 7.

7. Evidence Quality Weighting (Grey Literature vs. Peer-Reviewed)

Issue: The review includes 5 peer-reviewed journal articles and 5 postgraduate theses/dissertations. While including grey literature is excellent for mitigating publication bias, treating unpublished academic theses with the identical weight as peer-reviewed publications without adjusting the narrative synthesis can compromise robustness.

Recommendation: Please add a brief sensitivity discussion or narrative analysis comparing whether the overall compliance rates or documented biases significantly differed between the peer-reviewed papers and the unpublished dissertations.

8. Critical Misalignment Between Stated Objectives and Reported Results

Issue: A rigorous systematic review demands strict parallelism between the research objectives and the synthesized results. In this manuscript, there is a distinct structural mismatch.

1. While the objectives aim to assess regional compliance, the Results section merely lists individual study findings consecutively without aggregating or pooling the data into meaningful descriptive summaries (e.g., median compliance ranges).

2. The investigation of "determinants and barriers" promised in the framework is presented without a structured thematic coding system, making the narrative synthesis appear anecdotal rather than systematic.

3. Any comparative objective across different West African settings is entirely neglected in the results, despite the inclusion of both peer-reviewed papers and grey literature from two distinct countries (Ghana and Nigeria).

Recommendation: The authors must drastically restructure the Results section to mirror the specific objectives point-by-point. They should utilize explicit subheadings in the Results that correspond directly to each research question, and ensure that data from all 10 included studies are synthesized collectively under those specific themes rather than reported as isolated study summaries.

9. Superficial and Unstructured Discussion Section

Issue: The Discussion section is currently underdeveloped and mirrors a superficial repetition of the findings rather than offering a critical, contextualized synthesis. Because the Results lack a clear comparative and thematic structure, the Discussion fails to deeply interpret why OHS compliance rates and barriers differ or align between the two distinct nations (Ghana and Nigeria). It lacks a broader epidemiological and public health contextualization regarding the socio-economic, political, or legislative infrastructures governing the construction sectors in these specific West African settings.

Recommendation: The Discussion must be thoroughly revised. The authors should:

1. Move beyond merely restating the numbers and focus on interpreting the underlying systemic, institutional, or behavioral reasons behind the documented OHS compliance rates.

2. Provide a robust, critical comparison explicitly discussing how the differences in national occupational health policies, enforcement mechanisms, or socioeconomic contexts between Ghana and Nigeria could explain the variances in the study outcomes.

### Minor Concerns

1. Table Condensation and Formatting

Issue: Splitting the descriptive results into 5 separate, consecutive tables (Tables 3, 4, 5, 6, and 7) creates unnecessary fragmentation and heavily disrupts scannability. Furthermore, some cells and column boundaries contain truncated text or awkward spacing.

Recommendation: I strongly recommend consolidating Tables 3, 4, 5, and 6 into a single, cohesive "Domain-Specific OHS Compliance Findings" matrix table. This will allow readers to easily evaluate and cross-compare different compliance domains across the 10 included studies.

2. Abstract Refinement

Issue: The abstract mentions that the search ran through "February 2025", but lines 130 and 141 specify the absolute cutoff date as "12th February 2025".

Recommendation: Ensure absolute alignment of dates.

3. Unexplained Terminology

Issue: On line 239, the authors use the phrase "DIK1-classified contractors in Ghana". This localized classification system will not be understood by a global public health audience.

Recommendation: Provide a brief parenthetical clarification explaining what a "DIK1" classification signifies (e.g., large-scale/financial capacity tier) within the Ghanaian construction ecosystem.

Reviewer #2: Comments for revision

1. The title is confusing. It refers to Ghana, but the review includes Ghana and Nigeria. Manuscript repeatedly frames evidence as West Africa. A clearer title would be: “Occupational health and safety compliance among construction workers in Ghana and Nigeria: a systematic review of available quantitative evidence”

2. All included studies are cross-sectional. No intervention studies were included. Therefore, the review cannot assess effective strategies. It can only summarize reported or proposed strategies. Revise the objectives to synthesize quantitative evidence, and to identify factors associated with compliance. The research question should also be refined.

3. Search strategy from Google Scholar : State whether the first 100, 200, or all retrieved records were screened. Consider adding Scopus, Web of Science, or Embase, if accessible. If these were not searched, acknowledge this as a limitation.

4. The decision rule for exclusion must be fully described. The manuscript should state How JBI items were scored, what threshold defined low, moderate, and high risk of bias, which domains caused exclusion and whether excluding them changed the conclusions.

6. “Compliance” is inconsistently defined. The manuscript combines very different constructs under compliance: PPE use, PPE availability, employer provision, training attendance, knowledge, self-reported agreement with safety policies, inspections, and welfare facilities. These are not equivalent. For eg, “helmet access,” “helmet use,” “helmet borrowed from colleagues,” and “employer provision of helmets” measure different things. Similarly, “knowledge of PPE use” is not compliance. I recommend defining compliance operationally and separating outcomes

7. Numerical inconsistencies need checking. Table 3 reports Boakye et al. safety belt use as both 42.1% and 65.6%. This appears duplicative or inconsistent.

8. All included studies are cross-sectional and mostly self-reported. Hence the manuscript should avoid causal wording such as “leads to,” “results in,” or “root cause”.

9. The discussion should be balanced. Distinction should be made between evidence, inference, and recommendation.

**********

what does this mean?). If published, this will include your full peer review and any attached files.

**Do you want your identity to be public for this peer review?**  If you choose “no”, your identity will remain anonymous but your review may still be made public.

For information about this choice, including consent withdrawal, please see our Privacy Policy

Reviewer #1: **Yes:**  Phnom Sukchan

Reviewer #2: **Yes:**  Karthik Balajee Laksham

**********

[NOTE: If reviewer comments were submitted as an attachment file, they will be attached to this email and accessible via the submission site. Please log into your account, locate the manuscript record, and check for the action link "View Attachments". If this link does not appear, there are no attachment files.]

Attachment Submitted filename: Reviewer Comments.pdf

# Author comment: Author response to Decision Letter 1

**DOI:** 10.1371/journal.pgph.0006724.r002

3 Sep 2026

Attachment Submitted filename: Response to Reviewers.pdf

# Peer review: Decision Letter 1

**DOI:** 10.1371/journal.pgph.0006724.r003

**Authors:** Theingi Maung Maung

18 Oct 2026

PGPH-D-26-02159R1

Occupational health and safety standards compliance among construction workers in West Africa: A systematic review of available evidence from Ghana and Nigeria

PLOS Global Public Health

Dear Dr. Anim,

Thank you for submitting your manuscript to PLOS Global Public Health. After careful consideration, we feel that it has merit but does not fully meet PLOS Global Public Health’s publication criteria as it currently stands. Therefore, we invite you to submit a revised version of the manuscript that addresses the points raised during the review process.

Overall, the authors have addressed the previous comments constructively and comprehensively. However, the remaining internal inconsistencies should be corrected, and some wording requires further refinement.

Please submit your revised manuscript by Nov 17 2026 11:59PM. If you will need more time than this to complete your revisions, please reply to this message or contact the journal office at globalpubhealth@plos.org. When you're ready to submit your revision, log on to https://www.editorialmanager.com/pgph/ and select the 'Submissions Needing Revision' folder to locate your manuscript file.

Please include the following items when submitting your revised manuscript:

- A letter that responds to each point raised by the editor and reviewer(s). You should upload this letter as a separate file labeled 'Response to Reviewers'.
- A marked-up copy of your manuscript that highlights changes made to the original version. You should upload this as a separate file labeled 'Revised Manuscript with Track Changes'.
- An unmarked version of your revised paper without tracked changes. You should upload this as a separate file labeled 'Manuscript'.

Guidelines for resubmitting your figure files are available below the reviewer comments at the end of this letter.

As the corresponding author, your ORCID iD is verified in the submission system and will appear in the published article. PLOS supports the use of ORCID, and we encourage all coauthors to register for an ORCID iD and use it as well. Please encourage your coauthors to verify their ORCID iD within the submission system before final acceptance, as unverified ORCID iDs will not appear in the published article. *Only*  the individual author can complete the verification step; PLOS staff *cannot*  verify ORCID iDs on behalf of authors.

We look forward to receiving your revised manuscript.

Kind regards,

Theingi Maung Maung, Ph.D

Academic Editor

PLOS Global Public Health

Journal Requirements:

If the reviewer comments include a recommendation to cite specific previously published works, please review and evaluate these publications to determine whether they are relevant and should be cited. There is no requirement to cite these works unless the editor has indicated otherwise.

Please review your reference list to ensure that it is complete and correct. If you have cited papers that have been retracted, please include the rationale for doing so in the manuscript text, or remove these references and replace them with relevant current references. Any changes to the reference list should be mentioned in the rebuttal letter that accompanies your revised manuscript. If you need to cite a retracted article, indicate the article’s retracted status in the References list and also include a citation and full reference for the retraction notice.

Additional Editor Comments (if provided):

[Note: HTML markup is below. Please do not edit.]

Reviewers' comments:

Reviewer's Responses to Questions

**Comments to the Author**

Reviewer #1: All comments have been addressed

Reviewer #3: (No Response)

**********

publication criteria?

Reviewer #1: Yes

Reviewer #3: Partly

**********

3. Has the statistical analysis been performed appropriately and rigorously?

Reviewer #1: Yes

Reviewer #3: Yes

**********

4. Have the authors made all data underlying the findings in their manuscript fully available (please refer to the Data Availability Statement at the start of the manuscript PDF file)?

The PLOS Data policy

Reviewer #1: Yes

Reviewer #3: Yes

**********

5. Is the manuscript presented in an intelligible fashion and written in standard English?

Reviewer #1: Yes

Reviewer #3: Yes

**********

Reviewer #1: (No Response)

Reviewer #3: The revised manuscript has improved substantially and the authors have addressed most of the major concerns raised in the previous review. In particular, updating the search, clearly distinguishing the different compliance-related constructs, removing the review-generated composite compliance scores, undertaking a source-level verification of the extracted data, providing greater clarity around the JBI assessment and protocol deviation, and adding sensitivity analyses have strengthened the review considerably. The revised objectives, Results and Discussion are also better aligned with what can be supported by the available cross-sectional evidence.

I have a few remaining concerns that should be addressed before acceptance. Most importantly, there appear to be some remnants of the previous version in the revised manuscript. For example, the manuscript refers at one point to “120 methodologically sound studies,” although the primary synthesis includes 12 studies, and the old “available upon reasonable request” Data Availability statement also appears to remain alongside the revised statement. I would therefore recommend a careful final check of the entire manuscript for duplicated or outdated text and numerical inconsistencies.

I would also suggest avoiding the description of all included studies as “methodologically sound,” since the primary synthesis includes studies assessed as both low and moderate risk of bias. Similarly, the statement that the other 14 West African countries have no methodologically sound evidence may be too strong, particularly given the English-language restriction and omission of Scopus, Web of Science and Embase. It would be more appropriate to state that no eligible low- or moderate-risk studies from these countries were identified through the present review. The overall conclusion may also be better framed in terms of heterogeneous gaps across OHS compliance-related domains rather than describing compliance overall as “low to moderate,” since the revised manuscript appropriately recognizes that these domains cannot be treated as a single compliance construct.

Overall, the authors have responded constructively and comprehensively to the previous comments. Subject to correction of these remaining internal inconsistencies and some further refinement of the wording, I consider the manuscript substantially improved.

**********

what does this mean?). If published, this will include your full peer review and any attached files.

**Do you want your identity to be public for this peer review?**  If you choose “no”, your identity will remain anonymous but your review may still be made public.

For information about this choice, including consent withdrawal, please see our Privacy Policy

Reviewer #1: No

Reviewer #3: **Yes:**  AMRUTA N BANDAL

**********

[NOTE: If reviewer comments were submitted as an attachment file, they will be attached to this email and accessible via the submission site. Please log into your account, locate the manuscript record, and check for the action link "View Attachments". If this link does not appear, there are no attachment files.]

# Author comment: Author response to Decision Letter 2

**DOI:** 10.1371/journal.pgph.0006724.r004

22 Oct 2026

Attachment Submitted filename: Response to Reviewers 2.pdf

# Editor report: Decision Letter 2

**DOI:** 10.1371/journal.pgph.0006724.r005

**Authors:** Theingi Maung Maung

28 Oct 2026

Occupational health and safety standards compliance among construction workers in West Africa: A systematic review of available evidence from Ghana and Nigeria

PGPH-D-26-02159R2

Dear Miss Anim,

We are pleased to inform you that your manuscript 'Occupational health and safety standards compliance among construction workers in West Africa: A systematic review of available evidence from Ghana and Nigeria' has been provisionally accepted for publication in PLOS Global Public Health.

Before your manuscript can be formally accepted you will need to complete some formatting changes, which you will receive in a follow up email. A member of our team will be in touch with a set of requests.

Please note that your manuscript will not be scheduled for publication until you have made the required changes, so a swift response is appreciated.

IMPORTANT: The editorial review process is now complete. PLOS will only permit corrections to spelling, formatting or significant scientific errors from this point onwards. Requests for major changes, or any which affect the scientific understanding of your work, will cause delays to the publication date of your manuscript.

If your institution or institutions have a press office, please notify them about your upcoming paper to help maximize its impact. If they'll be preparing press materials, please inform our press team as soon as possible -- no later than 48 hours after receiving the formal acceptance. Your manuscript will remain under strict press embargo until 2 pm Eastern Time on the date of publication. For more information, please contact globalpubhealth@plos.org.

Thank you again for supporting Open Access publishing; we are looking forward to publishing your work in PLOS Global Public Health.

Best regards,

Theingi Maung Maung, Ph.D

Academic Editor

PLOS Global Public Health

***********************************************************

Reviewer Comments (if any, and for reference):
